# Mendelian and Non-Mendelian Regulation of Gene Expression in Maize

**DOI:** 10.1371/journal.pgen.1003202

**Published:** 2013-01-17

**Authors:** Lin Li, Katherine Petsch, Rena Shimizu, Sanzhen Liu, Wayne Wenzhong Xu, Kai Ying, Jianming Yu, Michael J. Scanlon, Patrick S. Schnable, Marja C. P. Timmermans, Nathan M. Springer, Gary J. Muehlbauer

**Affiliations:** 1 Department of Agronomy and Plant Genetics, University of Minnesota, Saint Paul, Minnesota, United States of America; 2 Cold Spring Harbor Laboratory, Cold Spring Harbor, New York, United States of America; 3 Department of Plant Biology, Cornell University, Ithaca, New York, United States of America; 4 Department of Genetics, Development, and Cell Biology, and Department of Agronomy, Iowa State University, Ames, Iowa, United States of America; 5 Supercomputing Institute for Advanced Computational Research, University of Minnesota, Minneapolis, Minnesota, United States of America; 6 Department of Agronomy, Kansas State University, Manhattan, Kansas, United States of America; 7 Department of Plant Biology, University of Minnesota, Saint Paul, Minnesota, United States of America; The University of North Carolina at Chapel Hill, United States of America

## Abstract

Transcriptome variation plays an important role in affecting the phenotype of an organism. However, an understanding of the underlying mechanisms regulating transcriptome variation in segregating populations is still largely unknown. We sought to assess and map variation in transcript abundance in maize shoot apices in the intermated B73×Mo17 recombinant inbred line population. RNA–based sequencing (RNA–seq) allowed for the detection and quantification of the transcript abundance derived from 28,603 genes. For a majority of these genes, the population mean, coefficient of variation, and segregation patterns could be predicted by the parental expression levels. Expression quantitative trait loci (eQTL) mapping identified 30,774 eQTL including 96 *trans-*eQTL “hotspots,” each of which regulates the expression of a large number of genes. Interestingly, genes regulated by a *trans-*eQTL hotspot tend to be enriched for a specific function or act in the same genetic pathway. Also, genomic structural variation appeared to contribute to *cis-*regulation of gene expression. Besides genes showing Mendelian inheritance in the RIL population, we also found genes whose expression level and variation in the progeny could not be predicted based on parental difference, indicating that non-Mendelian factors also contribute to expression variation. Specifically, we found 145 genes that show patterns of expression reminiscent of paramutation such that all the progeny had expression levels similar to one of the two parents. Furthermore, we identified another 210 genes that exhibited unexpected patterns of transcript presence/absence. Many of these genes are likely to be gene fragments resulting from transposition, and the presence/absence of their transcripts could influence expression levels of their ancestral syntenic genes. Overall, our results contribute to the identification of novel expression patterns and broaden the understanding of transcriptional variation in plants.

## Author Summary

Phenotypes are determined by the expression of genes, the environment, and the interaction of gene expression and the environment. However, a complete understanding of the inheritance of and genome-wide regulation of gene expression is lacking. One approach, called expression quantitative trait locus (eQTL) mapping provides the opportunity to examine the genome-wide inheritance and regulation of gene expression. In this paper, we conducted high-throughput sequencing of gene transcripts to examine gene expression in the shoot apex of a maize biparental mapping population. We quantified expression levels from 28,603 genes in the population and showed that the vast majority of genes exhibited the expected pattern of Mendelian inheritance. We genetically mapped the expression patterns and identified genomic regions associated with gene expression. Notably, we detected gene expression patterns that exhibited non-Mendelian inheritance. These included 145 genes that exhibited expression patterns in the progeny that were similar to only one of the parents and 210 genes with unexpected presence/absence expression patterns. The findings of non-Mendelian inheritance underscore the complexity of gene expression and provide a framework for understanding these complexities.

## Introduction

The maize species exhibits high levels of phenotypic variation, which is likely the result of both genetic and epigenetic variation [1]. Dissection of genetic and epigenetic variation may shed light on the understanding of phenotypic variation and provide tools to accelerate maize breeding. The maize genome has a complex organization with interspersed repetitive elements and genes [2]. The genomes of different maize inbreds can vary substantially due to single nucleotide polymorphisms [3], small insertions/deletions [4]–[5], gene copy number variation (CNV) and genomic presence-absence variation (PAV) [2], [6]–[7]. Transposable elements, discovered in maize by Barbara McClintock [8]–[9], comprise a significant portion of the maize genome [2], [10]–[13] and can contribute substantially to genomic variation among lines [14]–[17]. There are many examples illustrating the potential for transposons to capture and mobilize genes or gene fragments [14], [18]–[24]. In addition to genetic changes, there is also evidence for epigenetic variation among maize inbred lines. The epigenetic differences vary within maize populations and show relatively stable *trans-*generational inheritance [25]. These diverse forms of genetic and epigenetic variation likely interact to affect relative transcript abundance, which contributes to phenotypic variation among maize individuals.

Exploring transcriptome variation and elucidating the underlying mechanisms of transcriptional regulation may further our understanding of the molecular bases of phenotypic variation [26]–[27]. Several groups have used microarray profiling to compare the transcriptomes of maize inbreds [28]–[33]. A comparison of the F1 hybrids and the parents revealed that much of the parental variation resulted in additive expression with some rare examples of unexpected expression in the F1 [28], [33]. A recent RNA-seq based analysis of transcriptomic variation in 21 maize elite inbred lines found that a substantial number of genes showed presence/absence expression patterns [34].

Genetical genomics or expression quantitative trait loci (eQTL) mapping is an efficient method for understanding the genetic basis of transcriptome variation [26]–[27], [35]–[36]. eQTL mapping uses transcript abundance as a phenotypic trait and maps the genomic loci controlling the transcript abundance [35]. eQTL are generally classified as *cis-* or *trans-* depending on whether they are physically linked to the gene that is regulated or unlinked, respectively. Both *cis-* and *trans-*eQTLs have been identified in plants and while *trans-*eQTLs are more abundant, they generally explain less expression variation than *cis-*eQTLs [37]–[42]. Several eQTL mapping experiments have utilized microarrays to reveal the complexity of transcriptome variation and their underlying genetic regulators such as *trans-*eQTL hotspots in human, animals and plants [37], [39], [42]–[44]. eQTL mapping of transcriptome variation has also been employed directly to help dissect phenotypic variation [42], [45]–[46]. The analyses of transcriptome variation in segregating populations have generally focused on exploring how a single locus contributes variation to transcript abundance in a Mendelian fashion. However, there is also the potential for non-Mendelian segregation of gene expression levels [47].

RNA-based sequencing (RNA-Seq) provides several key advantages for transcriptome research including robust expression detection especially for lowly expressed genes, unprecedented access to the fine structure of the transcriptome, and powerful detection of all the transcripts not depending on the reference genome annotation [48]–[49]. Here, we employed RNA-Seq on shoot apices of a well-studied maize intermated RIL population derived from B73 and Mo17 (IBM) [50]. We characterized the relationship of transcriptional variation between the progeny population and the parents in detail to understand how the parental variation combines to affect transcript abundance. This analysis identified a number of genes that exhibit unexpected patterns of expression variation including paramutation-like segregation patterns and presence/absence expression patterns between progeny and parents. Meanwhile, global eQTL mapping, a pair-wise epistasis scan and co-expression analysis were conducted to dissect the possible factors underlying this variation.

## Results

**Figure 1:**
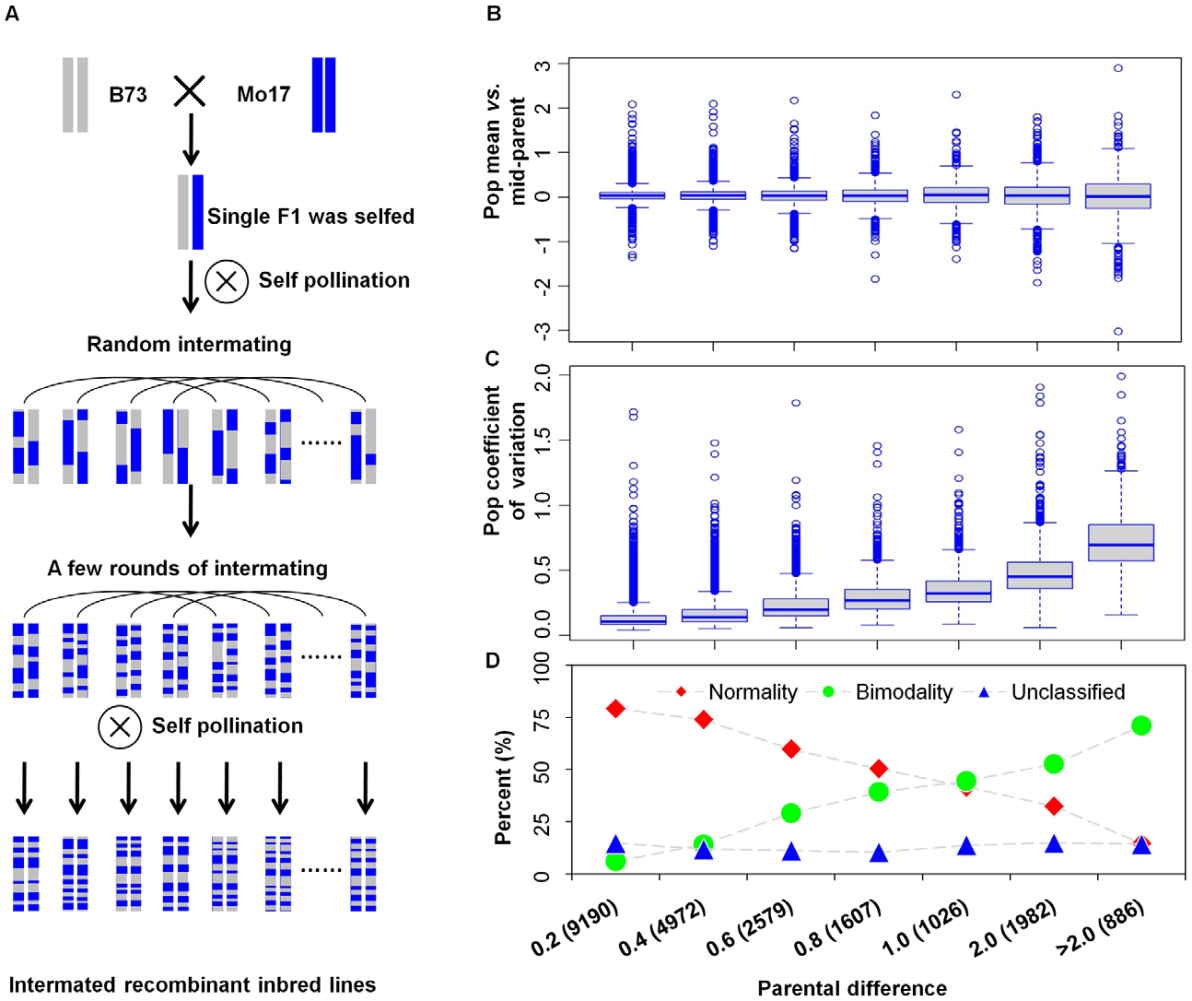
The intermated B73×Mo17 recombinant inbred lines (IBM RIL) and the relationship between expression variation in the RILs and expression fold change in the parents. (A) A schematic diagram of construction of the maize IBM RIL population (adapted from [50]–[51]). In (B), (C) and (D), the x-axis is the absolute value of log2 of expression-level in B73 divided by the level in Mo17. The numbers in parenthesis show the gene numbers in each category. (B) The expression-level relationship between the population mean and the parental difference. The y-axis is the log2 value of population expression-level average divided by the mid parent value of genes representing the expression-level deviation from the parents. (C) The coefficient of variation (CV) for gene expression levels in the RILs was significantly correlated with the parental differences. The y-axis shows the coefficient of expression-level variation of genes in the IBM RIL population. (D) The type of distribution observed in the RILs is influenced by the scale of parental difference. The proportion of genes that exhibit normal, bimodal or unclassified distributions of expression levels in the RILs vary according to the level of differential expression in the parental genotypes.

Global expression variation in maize was assessed using the intermated B73×Mo17 recombinant inbred line (IBM RIL) population [50]. The IBM RIL population provides higher genetic resolution than conventional RIL populations due to four generations of intermating before self-pollination (Figure 1A) [50]–[51]. RNA-seq was conducted on the shoot apices (4 mm cubic dissected tissue that includes the shoot apical meristem and several leaf primordia) of two-week old seedlings from the inbred lines B73 and Mo17, and 105 recombinant inbred lines (RILs) from the IBM population. In total, 3.47 billion reads (NCBI sequence read archive accession number-SRA054779) were obtained, trimmed for sequence quality and aligned to the B73 genome sequence v2 (AGPv2) [2]. For each genotype, an average of 23.5 million single end reads (93∼102 bp) were uniquely mapped to the annotated genes (Table S1). Based on the uniquely mapped reads, 28,603 genes were expressed in at least 10% of the RILs or at least one of the two parents at a false discovery rate of 0.05. A subset of 22,242 of these genes was expressed in both parents and in at least 90% of the RILs. Prior to further analysis, quantitative Real Time-PCR (qRT-PCR) was employed to assay the accuracy of the RNA-seq results by randomly selecting ten genes that exhibit a range of mean expression-levels. The qRT-PCR results largely confirmed the RNA-seq results, showing the accuracy of RNA-seq for RNA profiling () as in previous studies [48]–[49].

### Variation in gene expression levels in RILs

A population of RILs is expected to segregate 1∶1 for the parental alleles and provides an opportunity to examine variation in transcript abundance within the RILs and the relationship between the population and the parents. We first focused on the expression levels of 22,242 genes that were detected in both parents and at least 90% of the IBM RILs. The mean expression levels in the RILs were similar to the mid-parent values for most genes (Figure 1B). Transgressive segregation, defined here as at least 10% of RILs exhibiting expression levels outside the parental range, was observed for 598 genes (2.6%). The other 21,644 (97.4%) genes have expression levels in the RILs that are within the parental range. The level of variation for gene expression levels in the RILs was significantly correlated with the difference between the two parents (Pearson's product-moment correlation: *r* = 0.728, *P*<2.2E-16; Figure 1C). The type of distribution for expression levels within the RIL population relative to the parents was assessed using a τ score [52]. We found that 4,822 (22%) genes fit bimodal distributions, 14,564 exhibited normality (65%) and the remaining 2,856 (13%) showed other unclassified distributions. Genes with little or no expression difference among the parents typically exhibited a normal distribution in the RILs (Figure 1D). However, many genes with large expression differences among the parents exhibited a bimodal distribution among the RILs (Figure 1D). These trends indicated that much of the variation in gene expression levels in the RILs is reflective of differences present between the parents.

### Paramutation-like expression pattern in RILs

**Figure 2:**
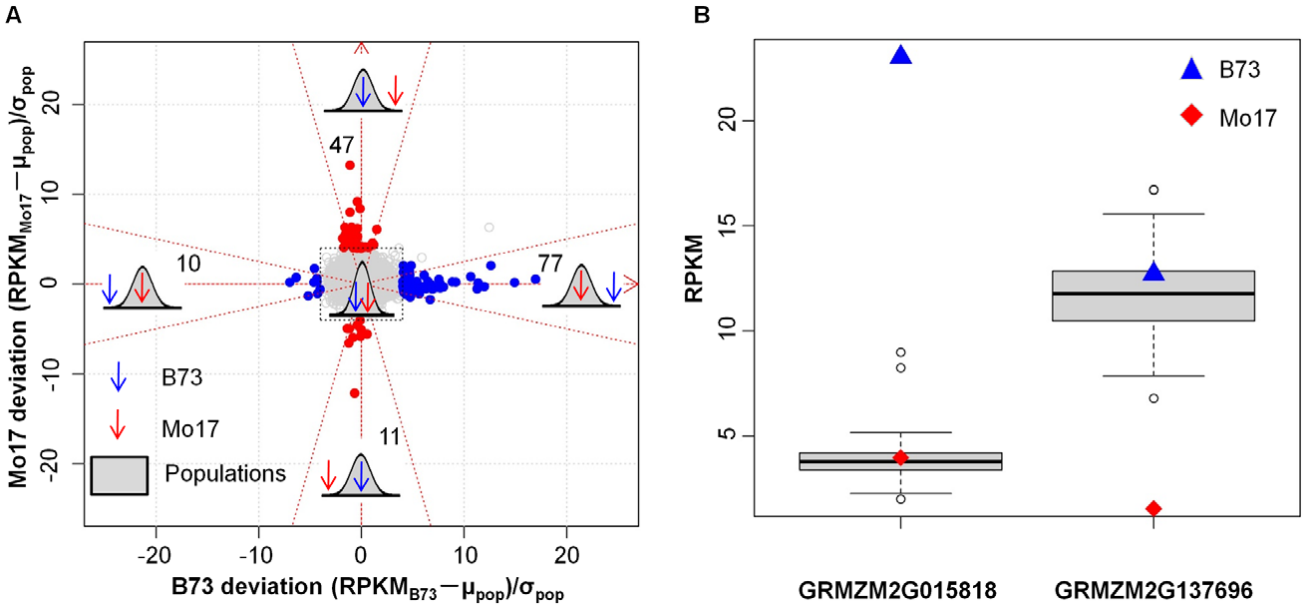
Paramutation-like expression patterns in the IBM RILs. (A) Two-dimensional representation of expression level variation among B73, Mo17 and the IBM RILs. The plot illustrates the expression level of B73 and Mo17 relative to the population mean and standard deviation for all 28,603 genes. The x-axis represents the number of standard deviations of difference between B73 and the RIL population while the y-axis represents the number of standard deviations between Mo17 and the population mean. Each point represents the expression relationship between the two parents and the RILs for one gene. The blue and red circles indicate genes with paramutation-like expression patterns in which B73 is at least three standard deviations outside the range of the RILs (blue) or Mo17 is at least three standard deviations outside the range of the RILs (red). The density plots provide a visual representation of each type of pattern that was identified. To provide better resolution for those genes with paramutation-like expression patterns, four genes, of which the parental expression levels were extremely out of the range of the expression levels in the RILs, were not plotted, but listed in Table S2. (B) The distribution of expression levels is shown for two genes with paramutation-like expression patterns. The y-axis shows the RPKM value for the normalized expression levels.

While the majority of genes exhibit expression patterns in the RILs that are quite predictable from the parental levels, there were a subset of genes (0.7%) that have average expression levels in the RILs that are greater than 2-fold different than the mid-parent, indicative of other potential patterns of expression variation. It is possible that some of these genes may have expression patterns similar to those observed for genes that are subject to paramutation such that the expression levels in all RILs would be similar to the expression level of one of the parents [53]. The distribution of expression patterns in the RILs was compared to the parental expression patterns for 8,269 out of 28,603 detected genes that have at least two-fold expression level difference between B73 and Mo17. There were 145 genes (86 of these genes are from the 22,242 genes expressed in both parents and 90% of the RILs) with paramutation-like expression patterns for the RILs in which one parent was within the expression distribution (two standard deviations from the population mean) of the RILs but the other parent had an expression level at least three standard deviations from the population mean (Figure 2A; Figure S2; Table S2). It is important to note that, while these genes exhibit patterns that are similar to those expected due to paramutation these genes may either be directly regulated by paramutation or be secondary targets that are influenced by another factor that is subject to paramutation. For many (80/145) of these genes one of the two parents had an expression level that was outside the range of all RIL genotypes. The expression levels of B73 and Mo17 relative to the population mean and standard deviation helps illustrate several trends observed for these genes (Figure 2A). The majority of these genes (124/145) had patterns in which the RILs were all expressed at levels similar to the lower parent as might be expected given that most examples of paramutation involve a paramutagenic allele that is expressed at lower levels than the paramutable allele (Figure 2B). The expression level for these genes was assessed in the F1 hybrid relative to the two parents (Figure S3). Well characterized examples of paramutation in maize include some examples of dominant expression in the F1 as well as other examples that do not exert effects until the F2 generation [54]–[57]. The genes that had high levels of expression in all RILs were expressed at additive levels in the F1. The genes that had expression levels similar to the lower parent included many examples of additive expression but also had a number of cases with partial to complete dominance in expression such that the F1 had levels more similar to the lower parent (Figure S3). Ten of the genes with paramutation-like patterns were selected for analysis in F2 individuals (Figure S4). Seven of the ten genes exhibited paramutation-like patterns in the F2 individuals and these include examples of both high and low expression.

### Mapping the basis of expression level variation

**Figure 3:**
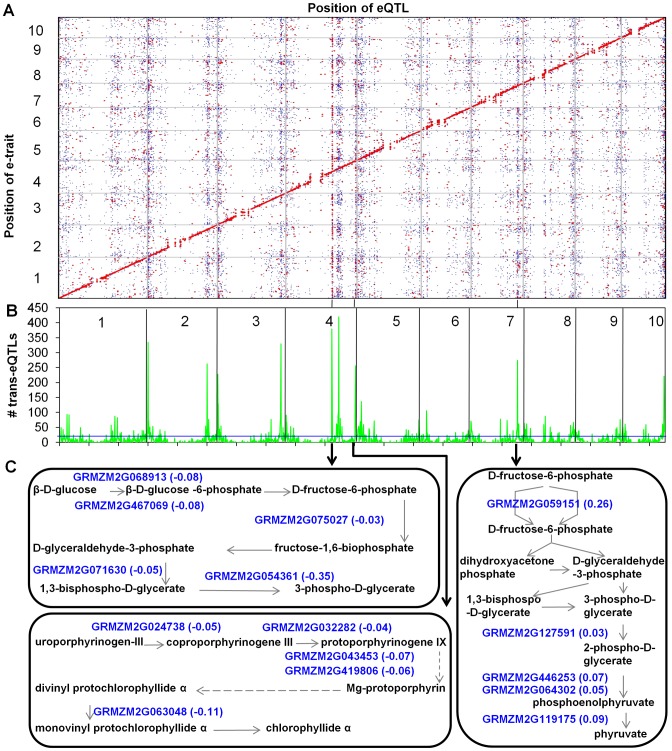
eQTL mapping, *trans-*eQTL hotspots, and pathways regulated by three *trans-*eQTL hotspots. (A) Genomic distribution of eQTLs identified in maize shoot apices. The x-axis indicates the genomic positions of eQTLs, while the y-axis shows the genomic positions of expressed genes (e-traits). The 10 maize chromosomes are separated by grey lines. The color of each point reflects the *R* ^2^ value. eQTLs with *R* ^2^ values greater than 20% were plotted in red, *R* ^2^ values less than 20% are indicated in blue. Totally, 30,774 eQTLs were divided into 11,504 (∼37%) *cis-*eQTLs and 19,270 (∼63%) *trans-*eQTLs. (B) The distribution of *trans-*eQTLs hotspots. The x-axis shows the genomic position of detected eQTLs (unit = 1 Mb), while the y-axis represents the number of *trans-*eQTLs in each 1 Mb length genomic region. The horizontal blue line for eQTL hotspots indicates the threshold, which is represented by the maximum number of *trans-*eQTLs expected to randomly fall into any interval with a genome-wide *P* = 0.01. The 10 maize chromosomes were divided by vertical black lines. The black lines linking (A) and (B) show several examples of the corresponding *trans-*eQTL hotspots in (A) and (B). A total of 96 *trans-*eQTLs hotspots were identified and 10 *trans-*eQTLs hotspots regulated at least 200 *trans-*eQTLs. (C) Genes regulated by three *trans-*eQTL hotspots are involved in specific metabolic pathways. The expression levels of these genes in pathways were regulated by *trans-*eQTLs located in these hotspots. The numbers beside these genes are the proportional changes which were the additive effects of the *trans-*eQTLs of Mo17 alleles divided by the population mean of expression levels of the target genes.

The basis for the regulatory variation in transcript levels was examined using a high-resolution SNP genetic map of the IBM population based upon 7,856 high quality SNP markers derived from the RNA-seq data to perform eQTL analysis for the 22,242 genes that are expressed in both parents and at least 90% of the RILs. This approach is likely to capture much of the variation for gene expression that segregates in a Mendelian fashion but is less likely to capture the basis of variation for examples of gene expression such as those described above. A total of 30,774 eQTLs (α = 0.05) with a threshold logarithm of odds (LOD)> = 4.17 were identified for 19,304 genes, of which 5,303 (27.5%) were controlled only by a single *cis-*eQTL, 6,201 (32.1%) controlled by both *cis-* and *trans-*eQTLs and 7,800 (40.4%) only by *trans-*eQTLs. The 30,774 eQTLs include 11,504 (∼37%) *cis-*eQTLs and 19,270 (∼63%) *trans-*eQTLs (Figure 3A and Table S3). The number of eQTLs affecting the expression level of each gene ranged from zero to six. In general, *cis-*eQTLs tend to have larger effects than *trans-*eQTLs (Figure S5A). For example, 83.7% of *cis-*eQTLs account for at least 20% of the expression variation in contrast to only 12.7% of the *trans-*eQTL meeting this criterion. However, there are examples of *trans-*eQTLs that contribute substantially to expression variation. There were 133 *trans-*eQTLs that contribute at least 60% of the variation for expression of a target gene. The overall contribution of *cis-* and *trans-*eQTLs was heavily influenced by the level of expression variation in the parents (Figure S5B). The contribution of *cis-*eQTLs increased as the parental expression level became increasingly different. In addition, the amount of variation explained by the *cis-*eQTL also increased as the parental expression levels become more different (Figure S5C) while the amounts of variation explained by *trans-*eQTL decreased as the parental differences increased (Figure S5D). The proportion of *cis-* and *trans-*eQTL for the 598 genes exhibiting transgressive segregation was similar to the proportion of *cis-* and *trans-*eQTL for the global eQTL analysis, however, the genes with transgressive segregation were more often (37%) controlled by multiple eQTLs with opposite effects than all genes (27%).

The genomic distribution of *trans-*eQTL was assessed in an attempt to identify potential *trans-*eQTL hotspots that might reflect substantial regulatory differences between B73 and Mo17. The analysis of *trans-*eQTL density in a 1 Mb (which is slightly larger than the average physical distance between adjacent markers with a recombination event) sliding window revealed 96 significant (*P*<0.01) *trans-*eQTL hotspots (Figure 3B and Table S4), including 10 major hotspots that contain at least 200 *trans-*eQTLs (Table 1). These hotspots have many more *trans-*eQTL than other genomic regions and in the majority (78%) of examples the target genes regulated at the *trans-*eQTL hotspots show a consistent pattern with significantly more target genes altered in expression in the same direction by the haplotype at the *trans-*eQTL hotspot (haplotype bias). More examples in which the B73 allele (49) at the *trans-*eQTL hotspot promoted higher expression of the target loci than the Mo17 allele (26) were identified. The lists of target genes regulated by each of the *trans-*eQTL hotspots were used to search for GO enrichments; 43% of the *trans-*eQTL hotspots target lists exhibited enrichments for at least one GO term (Table S5). We performed further analyses for the ten *trans-*eQTL hotspots that had at least 200 targets (Table 1). Nine of these ten *trans-*eQTL hotspots showed consistent haplotype bias (six for B73 and three for Mo17) and the targets for each of these hotspots had GO enrichments for at least one term. Multiple genes in the same MaizeCyc pathway [58] are observed to be co-regulated by the same *trans-*eQTL hotspot (Figure 3C, Table S6). These *trans-*eQTL hotspots may be due to functional differences in transcriptional regulators. At least in some cases it might be expected that differential expression of a regulator present at the *trans-*eQTL hotspot is the cause of the differences in *trans-*regulation.

**Table 1:** *Trans-*eQTL hotspots with at least 200 *trans-*eQTLs.

Hotspot_name	Chr	StartPos (Mb)	EndPos (Mb)	#_*cis* a	#_*trans* b	#_eQTL/(Mb×#_genes)	B73^c^	Mo17^d^	Sig. Bias^e^	GO Term enrichment	MaizeCyc enrichment
*Zm_eQTL_HS14*	2	3	5	56	353	3.18	289	64	4.77E-33	Yes	No
*Zm_eQTL_HS20*	2	202	206	70	263	2.10	161	102	2.75E-04	Yes	No
*Zm_eQTL_HS25*	3	4	6	28	228	3.51	110	118	5.96E-01	Yes	No
*Zm_eQTL_HS29*	3	214	218	63	336	2.95	87	249	9.76E-19	No	No
*Zm_eQTL_HS35*	4	157	160	30	379	5.92	321	58	1.38E-41	Yes	Yes
*Zm_eQTL_HS37*	4	176	182	45	420	2.80	274	146	4.22E-10	Yes	Yes
*Zm_eQTL_HS41*	4	236	238	38	259	2.78	242	17	2.04E-44	Yes	Yes
*Zm_eQTL_HS60*	7	0	2	22	162	2.57	119	43	2.36E-09	Yes	No
*Zm_eQTL_HS65*	7	156	160	51	274	2.14	82	192	3.03E-11	Yes	Yes
*Zm_eQTL_HS95*	10	145	147	35	221	2.83	64	157	3.95E-10	Yes	Yes

a,b Indicates the number of *cis-* and *trans-*eQTLs in each eQTL hotspot, respectively.

c Indicates the number of eQTLs, where the B73 allele increased the expression level in the RIL population.

d Indicates the number of eQTLs, where the Mo17 allele increased the expression level in the RIL population.

e Shows the significance level deviating from the random distribution between B73 and Mo17. The GO enrichments and the pathway enrichments of the regulated genes by hotspots were conducted using BiNGO plugin in Cytoscape based on the annotation information from AgriGO and MaizeCyc database, respectively. The results of GO enrichments and pathway enrichments are in Table S5 and Table S6, respectively.

### Structural variants associated with the regulatory variation

**Figure 4:**
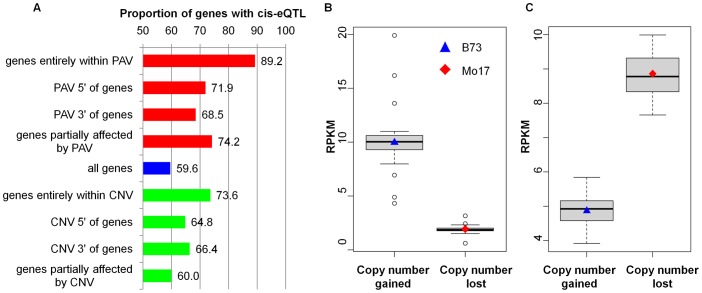
eQTL with CNV/PAV nearby and the influence of CNV/PAV on transcriptome variation. (A) The proportion of genes with *cis-*eQTL detected. Genes located within/near structural variants are enriched for *cis-*eQTLs, especially for genes entirely within CNV/PAV. (B) The expression distribution in the RILs of gene *GRMZM2G016150*, which is entirely a CNV event, is positively correlated (*P* = 3.7E-46) to increased copy number at this locus in the RILs. (C) The expression distribution in the RILs of gene *GRMZM2G024775*, which exhibits a gain of a copy in B73 and lines containing the B73 allele, shows a negative correlation (*P* = 6.3E-61) between the gain of a copy in B73 and the lower copies in Mo17. In (B) and (C), the x-axis represents the genotype of RILs for the specific gene, while the y-axis indicates the normalized expression levels in the RILs and their parents.

To examine the influence of structural rearrangements-gene copy number variation (CNV) and genomic presence/absence variation (PAV) on gene expression, we compared our transcriptomic data for the 28,603 expressed genes with previous Comparative Genomic Hybridization (CGH) data [59]. We focused on the full set of 28,603 genes as the more limited set of 22,242 genes assessed for eQTL analysis required expression to be present in both parents while some of the PAV are expected to abolish expression in Mo17. There are 1,212 expressed genes with CNV/PAVs that affect the gene or flanking regions (Table S7). The structural rearrangements include copy number gains in B73 or Mo17 as well as PAV that are present in B73 but absent in the Mo17 genome. We might expect that copy number gains would lead to increased expression in the genotype with more copies while PAV would only be expressed in one genotype. There was evidence that this was true in many cases (Table S8 and Figure 4). eQTL mapping was conducted on these CNV/PAV-related genes and a total of 1,466 were identified for 1,009 genes, of which 704 (69.8%) were controlled by *cis-*eQTLs. The *cis-*eQTLs proportion of genes with CNV/PAVs nearby is significantly higher (*P* = 0.00) than those of all detected genes (Figure 4A). Noteworthy was the observation that 89.2% of these genes entirely within the PAV were controlled by *cis-*eQTLs, while ∼10% of these genes have *trans-*eQTLs, indicating that other regulators underlie the expression variation in addition to PAVs. There was also evidence for an enrichment of *cis-*acting variation when the CNV/PAV occurred in regions surrounding the gene. Nearly half (120/242) of the genes entirely within structural variants exhibit differential expression in B73 and Mo17. There were many examples in which the RIL genotype at the gene of interest was highly correlated with the expression difference (Figure 4B, 4C). Typically, the copy number of genes entirely within CNV/PAV regions positively correlated with the genes' expression (99 out of the 120 differentially expressed genes between the two parents) (Figure 4B). We also noted examples (21/120) in which a copy number gain was associated with lower expression in the parents (Figure 4C).

### Unexpected patterns for presence/absence expression in the RILs

We were struck that a large proportion of genes were only detected in a subset of the RILs or parents. While there were 22,242 genes expressed in both parents and the RILs, there were an additional 6,361 genes that had detectable (False Discovery Rate-FDR>0.05) levels in at least 10% of the RILs or at least one of their parents. These 6,361 genes may include (a) some genes with very low expression levels that manage to cross the threshold of detectability in some samples but not others, (b) genes that are only expressed in one parent and that based on Mendelian segregation would therefore be expected to be expressed in only 50% of the RILs, and (c) genes with unusual regulatory mechanisms. We elected to impose a more restrictive set of filtering criteria for expression to limit the number of low-expressed genes near the detection threshold. Based on the alignment of RNA-seq reads to non-genic genomic regions, an RPKM of 1.03 corresponds to a FDR of 0.01 and 499 of the 6,361 genes have a RPKM value of ≧1.03 in at least 10% of the RILs or at least one of their parents. A substantial proportion of these genes (289/499) were expressed in only one of the parents and were observed in approximately 50% of the RILs (with the Chi-square test at the *P* value<0.01). The lack of expression in one parent and half of the RILs may reflect differences in genome content or regulatory variation. eQTL analysis of these genes revealed that 186 (64%) of these genes had *cis-*eQTL that explained >20% of the expression variation and 54 of these genes intersect with CNV/PAVs. However, there were also 92 (32%) of these genes that had evidence for at least one strong *trans-*eQTL with *R* ^2^>20%. In total, eQTLs could explain more than 20% of the expression variation of 273 of these 289 genes (96.1%).

**Figure 5:**
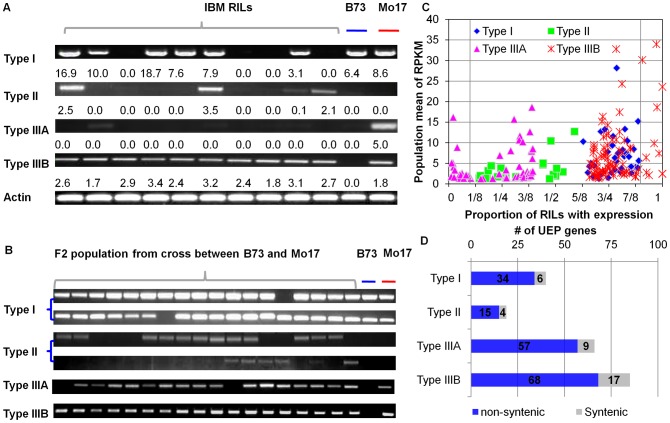
Four types of unexpected expression patterns between the RILs and their parents. Type I: genes expressed in both B73 and Mo17, but not expressed in at least 1/10 of the RIL population. Type II: genes not expressed in both B73 and Mo17, but expressed in at least 1/10 of the RIL population. Type III: genes expressed in either B73 or Mo17, have abnormal segregation ratio of expression versus non-expression in RILs, such as 1∶3, 3∶1 etc. Type IIIA are genes tending to be expressed in fewer RILs than the expected 1∶1 ratio while Type IIIB are genes that tend to be expressed in more RILs than the expected 1∶1 ratio. In (A), gene transcripts in the Type I∼III categories were amplified by RT-PCR from RNAs isolated from an independent replication of 10 genotypes from the IBM population. Thirty-five cycles of PCR was conducted for genes *GRMZM2G403162* (Type I), *GRMZM2G168987* (Type II), *GRMZM2G103479* (Type IIIA), *GRMZM2G170588* (Type IIIB) and a housekeeping gene (*Actin*). The number under each band shows the RPKM value in each RIL. (B) RT-PCR assay of individuals in an F_2_ population from the cross between B73 and Mo17. The corresponding genes from top to bottom are *GRMZM2G403162*, *GRMZM2G053790*, *GRMZM2G168987*, *GRMZM2G071808*, *GRMZM2G103479*, and *GRMZM2G170588*. (C) The percent of RILs with expressed genes with unexpected expression patterns and population mean of their expression levels in the RILs. The x-axis represents the percent of RILs, while the y-axis indicates the log2 score of the population mean of RPKM. The two grey vertical lines mark 10% and 90% of the RILs. (D) The number of genes for each of these unexpected expression patterns and the proportion of syntenic and non-syntenic genes in each expression pattern.

The other 210 of these genes exhibited unexpected patterns of expression that could be classified into four groups (Table S9). The type I pattern included 40 genes that were expressed in both parents but were not detected (RPKM = 0) in over 10% of the RILs. The type II pattern included 19 genes that were not detected (RPKM = 0) in the parents but were detected in at least 10% of the RILs. The type III patterns include genes that were expressed in one parent but not the other and had expression in very few RILs (type IIIA – 66 genes) or the majority of the RILs (type IIIB – 85 genes) (Figure 5A). A subset of genes (2 type I genes and 19 type III genes) with unexpected expression patterns also exhibited paramutation-like expression patterns. These unexpected patterns of expression detected by RNA-seq were validated for the majority of genes tested (43/55) using RT-PCR on a subset of the RIL genotypes (Figure S6). In addition, the same type of expression patterns could be observed in an independent set of B73×Mo17 F2 individuals for all the six tested genes (Figure 5B). These RT-PCR assays confirmed that the unexpected segregation patterns for presence or absence of gene expression observed in the RILs are reproducible. Further, genomic PCR was employed to assess if the expression presence/absence transcript variation might be attributable to differences in genome content. We found that genes exhibiting presence/absence transcript variation could be amplified from genomic DNA of each of the IBM RILs that were tested (Figure S7), indicating that the difference in expression was not due to segregation for genomic presence of the sequence. For each of the four patterns, the proportion of RILs expressing a gene was compared to the mean expression level in genotypes that express the gene (Figure 5C). Some of these genes are quite highly expressed and there is a substantial range in the number of genotypes with expression. To further distinguish the genes with unexpected expression patterns from the genes with very low expression levels that manage to cross the threshold of detectability, we examined the maximum expression levels, population mean RPKM and the standard deviations in the population of the genes with unexpected expression patterns in comparison to all detected genes expressed in more than 90% of the RILs. Although the maximum expression levels, population mean RPKMs of genes with unexpected expression patterns are slightly lower than those of all expressed genes, the differences are not significant (Figure S8). Importantly, the standard deviation of expression levels of genes with unexpected expression patterns is similar to that of all other genes (Figure S8).

The observation that there were many examples in which the proportions of RILs with detected expression was close to 25% or 75% (Figure 5C) may suggest that multiple genetic factors play interaction roles underlying the unexpected expression patterns for some of these genes. To test this hypothesis, a genome-wide epistasis scan with all possible pair-wise marker interactions was employed to search for evidence of two-locus interactions that control expression for genes that were detected in approximately one-quarter or three-quarters of the RILs. If two different loci are both required to achieve expression of a gene, these loci could both be present in one parent (type III) or could have one functional locus in either parent (type II). In these examples we would expect 25% of the RILs to exhibit expression of the gene. There are 28 type IIIA and 10 type II genes with expression in only ∼25% of the RILs using Chi-square test with the *p*-value<0.01 as the cut-off. A genome-wide scan for two-locus interactions that control the variation of expression for these 38 genes found that 92% of these could be explained by a two locus interaction (Figure S9A, S9B). In half of the cases in which a two-locus interaction explained a significant proportion of the expression variation we found that one of the two loci mapped in *cis* to the gene itself. We could also envision a scenario in which two different loci are required for loss of expression of a gene and this would be expected to result in expression in 75% of the RILs. There are 71 type IIIB and 28 type I genes that are expressed in ∼75% of the RILs and for 91% of these genes the pattern of presence/absence can be explained by a two-locus interaction, including 12 examples in which one of the two loci maps in *cis* to the gene itself (Figure S9C, S9D). This suggests that a significant subset of the genes with unexpected patterns of presence-absence for expression can be explained by two-locus interactions.

### Non-syntenic genes enriched in the genes with unexpected expression patterns

The genes that exhibit presence/absence expression patterns in progeny relative to their parents were further characterized. As a group, these genes with unexpected expression patterns were enriched for single copy genes, and for low copy number gene families relative to all maize genes (Table 2). The FGS (Filtered Gene Set) genes of maize represent an attempt to identify higher confidence gene models and remove gene fragments and transposon-derived sequences [2]. However, there are likely a number of gene fragments and transposon-derived sequences still present within the FGS. Comparative genomic localization can provide more confidence in syntenic genes as “real” genes [60]. Only 36/210 genes with presence/absence expression patterns are in syntenic locations relative to other grass species (Figure 5D). This is a smaller proportion than expected based on the finding that 67.5% of all FGS genes are located in syntenic positions. It is worth noting that while the genes with unexpected patterns are enriched for non-synteny there is a subset of these genes that do have synteny and likely represent functional genes (Table S9). Annotation of the syntenic genes with unexpected presence/absence expression patterns reveal a variety of putative functions such as serine threonine protein kinase, electron transport sco1 family protein and basic leucine-zipper 44 protein, but there is no evidence for GO enrichments within this set of genes.

**Table 2:** Gene family size for genes with unexpected expression patterns.

Genes	Count	Single Copy (%)	Gene family size (%)				Average gene family size
			2	3–6	7–10	>10	
Type I	40	35.0***	22.5***	25	2.5***	15.0***	8.42
Type II	19	26.3***	26.3***	15.8**	15.8*	15.8***	5.50*
Type IIIA	66	39.4***	22.7***	24.2	3.0***	10.6***	5.28***
Type IIIB	85	34.1***	10.6	40.0***	8.2	7.1***	5.30***
All maize	39400	18	13	28.9	10.8	29.3	8.64

*,**,*** represent P<0.05, P<0.01, and P<0.001, respectively. The significances were calculated by 10,000 permutations of randomly selected genes of which the gene number is equal to each expression pattern.

### Genes with unexpected expression patterns are likely to be transposon-related genes

**Figure 6:**
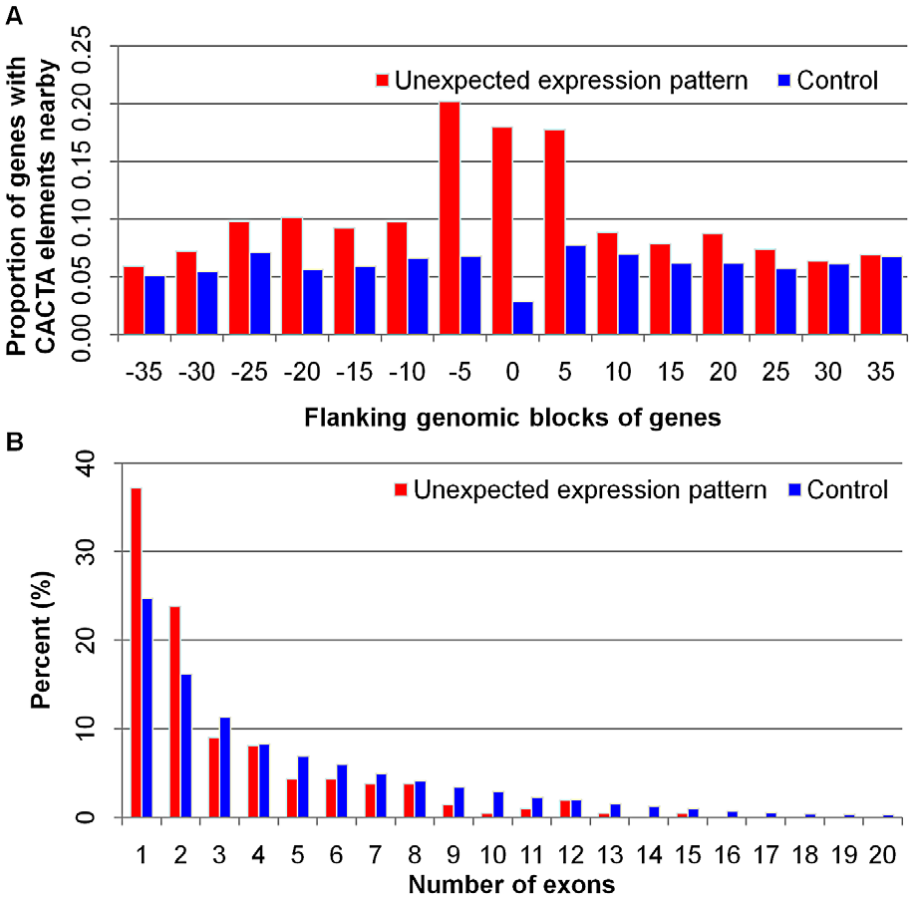
Enrichment of CACTA-like elements and fewer exon number bias in genes with unexpected expression patterns. (A) The proportion of genes with a CACTA-like element in different flanking genomic blocks. (B) Genes with unexpected expression patterns preferentially contain fewer exons.

The 174 genes with unexpected segregation patterns that are non-syntenic with other grass species may represent insertions of these genes or gene fragments in the maize genome. To test the hypothesis, the genomic regions surrounding these genes were examined for enrichment of specific classes of repetitive sequences (Figure S10). Over one-third (65) of the 174 genes had a CACTA-like element within 20 kb and these include examples of all types of unexpected expression patterns. This is significant (*P* = 0.00) enrichment of CACTA-like transposable elements surrounding these genes relative to the expected genomic frequency (Figure 6A). The 65 genes with CACTA-like sequences nearby (3.20 exons) and the other 145 genes with unexpected segregation patterns (3.10 exons) tended to have fewer exons (*P* = 0.00) than the average exon number (4.88 exons) of all maize genes (Figure 6B). These features, less exons, non-syntenic genomic localization and CACTA-like element enrichment, suggest that many of these genes may be gene fragments that were captured and transposed by CACTA-like transposons.

### Transposon-related genes with unexpected expression patterns could regulate the expression of their ancestral syntenic genes

**Figure 7:**
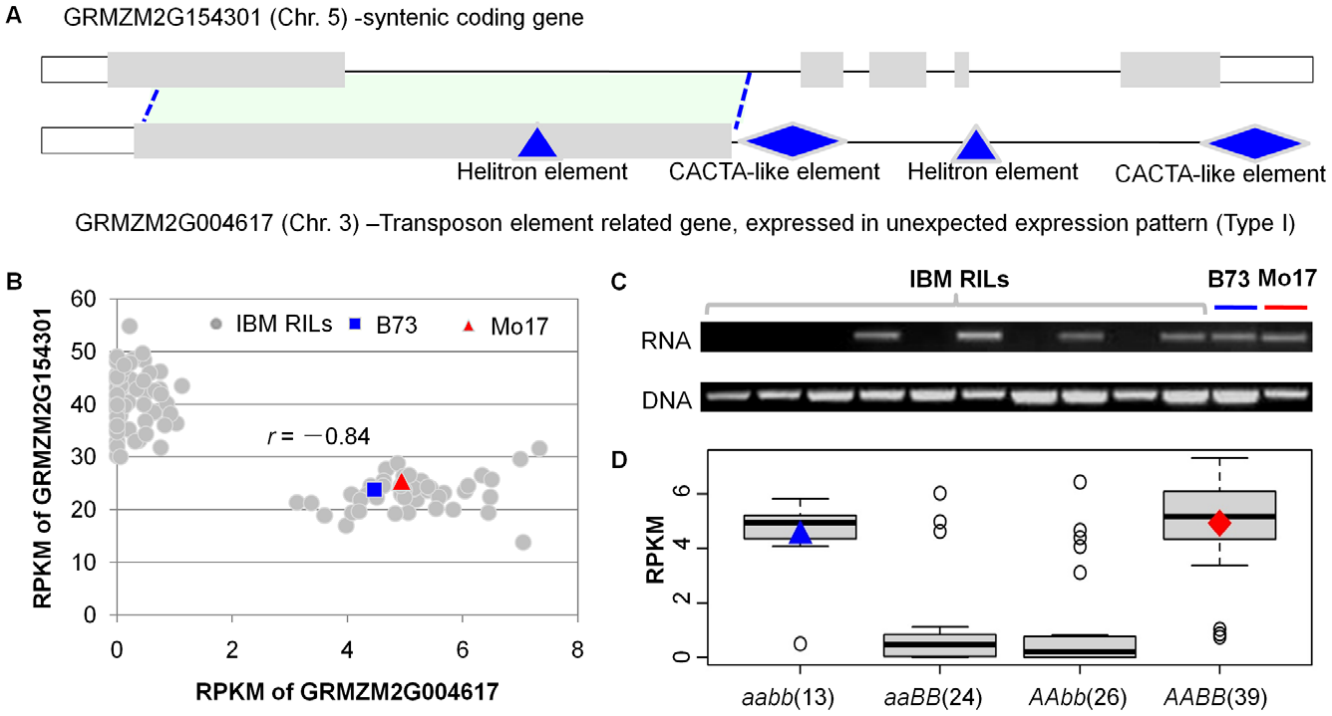
Co-expression complementary effect between a transposon-related gene and its ancestral syntenic gene. (A) The homologous relationship between the transposon-related gene (*GRMZM2G004617*) and its ancestral syntenic gene *GRMZM2G154301*. The blue dotted lines and the light blue area show the homologous region between the two genes. The grey boxes represent the coding regions, while the open boxes indicate the untranslated regions. The transposon annotation was done using CENSOR (http://www.girinst.org/censor/index.php). The blue triangle and diamond represent helitron element and CACTA-like element, respectively. (B) The negative co-expression correlation between the two genes. (C) The validation of RT-PCR and genomic DNA PCR of the transposon-related gene (*GRMZM2G004617*), which exhibits unexpected expression patterns in the RILs. (D) Two-marker interaction (M3735 located in chromosome 4 is designated as *locus A* and M5604 in chromosome 7 is denoted as *locus B*, neither of which are linked to the differentially expressed genes) was significantly associated with the transcriptomic variation of type I gene *GRMZM2G004617* in the RILs (*P* = 1.3E-07). *aabb* shows the genotype of B73, while *AABB* represents Mo17 genotype. The numbers close to the genotype show the number of RILs with the same genotype. The y-axis shows the normalized expression levels (RPKM). The blue triangle indicates the expression level in B73, while the red diamond indicates the expression level in Mo17.

We proceeded to assess whether the non-syntenic gene fragments with presence/absence expression might affect the regulation of homologous full-length syntenic genes (ancestral syntenic genes) elsewhere in the maize genome. All 174 of the non-syntenic genes were homologous to at least a portion of another maize gene (*E value*<1.0E-10). The correlation between the expression level of each of these genes and the other homologous full-length sequences (possible ancestral syntenic genes) was assessed in the RIL population. There were 25 examples in which the presence/absence expression patterns of the non-syntenic genes were correlated with transcript abundance for ancestral syntenic genes (Table S10). For example, the presence/absence expression of a gene fragment located on chromosome 3 was highly correlated with the abundance of a transcript from its ancestral syntenic gene annotated as an *Erwinia Induced Protein 1* located on chromosome 5 (Figure 7A). A comparison of the expression levels for the two sequences revealed an inverse correlation such that the presence/absence of transcripts from the gene fragment correlated with low or high expression of the ancestral syntenic gene (Figure 7B). However, the presence/absence of transcripts from the transposed fragment does not result from genomic differences among RILs because according to the genomic PCR amplifications this gene fragment exists in all tested RILs (Figure 7C). The expression pattern of gene *GRMZM2G004617* was also identified to be controlled by two-locus interaction (Figure 7D). Many (20) of the other 25 examples involve similar negative correlations between presence/absence of a gene fragment and abundance of a full-length transcript (Table S10). These examples provide evidence for the ability of transposed gene fragments to influence transcript abundance of their ancestral syntenic genes.

## Discussion

We used RNA-Seq to profile the shoot apex transcriptome variation within the maize IBM RIL population and to compare this variation to the parental B73 and Mo17 transcriptomes. In our study, we revealed that: (*i*) Much of the variation (the population mean, the coefficient of variation) in gene expression levels in progeny is reflective of differences present among the parents; (*ii*) A genome-wide search for paramutation-like expression identified 145 genes with paramutation-like patterns in the progeny; (*iii*) Multiple genes in a pathway are regulated in the same direction by a *trans-*eQTL hotspot, indicative of transcriptional regulators; (*iv*) CNV/PAVs could be either positively or negatively correlated with expression level variation; (*v*) A set of 210 genes were identified that exhibited unexpected presence/absence expression patterns within the RILs relative to the parents; and (*vi*) These genes with unexpected presence/absence expression patterns in the RILs likely include functional genes as well as transposed gene fragments that may contribute to regulatory variation of their ancestral syntenic genes. These findings provide an insightful understanding of the mechanisms that contribute to transcriptome variation in maize populations. We will discuss the identification of *trans-*eQTL hotspots and the implications for the unexpected segregation patterns of gene expression.

### *Trans-*eQTL hotspots

The analysis of eQTLs allows for the dissection of the genomic regions that affect transcript abundance. *Cis-*regulatory eQTL reflect regulatory variation that is tightly linked to a gene and affects the allelic expression levels. In contrast, *trans-*eQTL reflects regulatory variation at unlinked genomic positions. The analysis of all *trans-*eQTL can reveal *trans-*eQTL hotspots, also known as *trans-*eQTL clusters, which are genomic regions that affect the expression of many unlinked loci [39], [61]. These *trans-*eQTLs are thought to reflect differences in gene regulation that may be important for phenotypic variation [39], [41]–[42], [44].

Due to the limitations of mapping resolution, the identified *trans-*eQTL hotspots could result from the presence of a single causal regulatory factor (pleiotropic effects) or several tightly linked loci that affect transcript levels of different genes (genetic coupling) [62]. In addition, each *trans-*eQTL hotspot is relatively large (∼1 Mb) and will likely include the targets of the hotspot itself as well as several other *trans-*eQTLs that only regulate a small number of genes. Most of the *trans-*eQTL hotspots identified in our study showed significant haplotype effect bias, which means the haplotype of one parent could increase expression levels of significantly more target genes than expected. The hotspots with haplotype effect bias are more likely to reflect “master regulators”, while some of the others may be a result of genetic linkage, even though we had already taken gene density into account. It might be expected that variation in an important regulatory locus may result in variation for transcript levels for a number of genes that share related GO annotation or are present in the same biochemical pathway. Here, the expression level of genes involved in these pathways were found to be consistently altered in the same direction by *trans-*eQTL hostpots, which implies that pathway variation may exhibit genetic variation underlying the phenotypic variation among different elite inbred lines.

The regulatory variation provided by the *trans-*eQTL could be the result of differences in the expression level for a regulator located within the *trans-*eQTL (a *cis-*eQTL) or it could be the result of a qualitative variant for a gene located within the genomic region. If the cause of the *trans-*eQTL hotspot is a *cis-*regulatory variant then we would expect to find a *cis-*eQTL located within the *trans-*eQTL that is highly correlated with the expression level of the target genes. The analysis of these *cis-*eQTLs located within the *trans-*eQTL hotspot did not find enrichment for transcription factors. However, we did identify transcription factors or other putative regulatory genes. These candidate genes provide a potential avenue for future research to understand the basis of regulatory variation in maize (Table S11).

### Transgressive segregation for eQTL

The majority of genes behave in a manner that is predictable based on the expression levels of the parents. In general, genes with relatively little expression variation in the parental genotypes exhibit a normal distribution of expression levels centered on the parental levels in the offspring and the genes with variation between the parents exhibit a bimodal distribution in the offspring. Our results showed that for the majority of genes expression trait variation is mainly caused by additive effects, which differs from the results observed in *Arabidopsis*, and rice where non-additive gene action was the more common form of regulating transcript accumulation [39]–[42].

However, a portion of genes exhibit transgressive segregation in the RILs such that at least 10% of the RILs exhibit expression levels outside the parental range. The proportion of transgressive segregation for expression traits was small (2%) compared with the levels reported in other species [39]–[42]. The measurement of eQTL for many genes at once provides an opportunity to assess the potential causes of transgressive segregation. One likely cause of transgressive segregation would be the presence of multiple *trans-*eQTL including examples in which both parental haplotypes promote expression. For example, if a single gene has two *trans*-eQTLs for which the B73 allele promotes higher expression and two other *trans*-eQTLs in which the Mo17 allele promotes higher expression then one might expect to observe a number of RILs with expression levels that are higher or lower than the parental values due to segregation of these *trans*-eQTLs. Indeed, we found that the 598 genes with transgressive segregation tended to have higher numbers of *trans-*eQTL than the other genes and that these frequently included a mixture of B73/Mo17 favorable alleles for the underlying gene expression trait.

### Unexpected patterns of gene expression in off-spring relative to parents

While the majority of genes behaved in predictable fashions in the RILs relative to parents and had variation that could be attributed to eQTL there were some genes with unexpected expression patterns. We focused our analysis on a couple of subsets of these genes including genes with paramutation-like pattern of expression and genes with unexpected patterns of presence/absence of the transcripts.

When two parents exhibit variation in a trait it would be expected that off-spring would exhibit a similar range of variation. However, we found a number of genes for which none of the recombinant off-spring had expression levels similar to one of the parents. This is an apparent violation of Mendel's principle of segregation and might be reminiscent of paramutation. Paramutation describes instances in which there is communication between two alleles that are present in a heterozygote [53], [63]–[65]. The paramutable allele can be altered to behave more like a paramutagenic allele. Most of the examples of paramutation have been described in maize [64]. These examples include a variety of stabilities and behaviors [64] but are often sensitive to mutations in the same genes [65]–[67]. It has been hypothesized that paramutation will affect numerous other genes but that these other examples may not have been noted due to the lack of observable phenotypes. A recent study in tomato identified several transcripts that had expression patterns in RIL genotypes that were not indicative of the parental levels and could indicate paramutation [47]. We searched for examples of genes that had expression patterns that might be expected to result from paramutation. There were 145 examples of genes for which all of the RILs had expression similar to one of the parents while the other parent had a unique expression pattern. The majority (55%) of these genes represent examples in which the RILs all had expression levels similar to the lower expressing parent. The fact that these patterns were observed in RILs that have been subjected to >6 generations of inbreeding would suggest that these patterns of expression are relatively stable. While we do not have evidence to show direct interaction of the alleles in the heterozygote, we propose that the expression patterns observed for many of these 145 genes are the result of paramutation-like phenomena. Our analysis of expression in a RIL population relative to the parents suggests that paramutation-like mechanisms may contribute to regulatory variation for a number of maize loci. The analysis of F2 individuals provided further evidence for paramutation-like patterns for seven of the ten genes tested. It is possible that some of these examples may reflect spontaneous mutation or epimutation in the specific B73 and Mo17 individuals that were used for this study and these may account for the lack of validation for some examples. We also examined our dataset for genes whose expression was only detectable in a subset of the RIL population or at least one of the parents. Nearly 500 genes with various patterns of segregation for the presence/absence of transcripts were identified using a relatively stringent (FDR = 0.01) expression threshold. If the threshold for detection was relaxed (FDR = 0.05), the number of genes with segregation for presence/absence of transcripts increased to 4,689. These results suggest the presence of substantial qualitative as well as quantitative variation for the maize transcriptome following segregation. We further evaluated these genes to begin to understand the causes and consequences for this variation.

The most likely cause for variation in presence/absence of a transcript would be examples in which one parent expresses a gene and the other parent does not. In these instances we would expect approximately 50% of the RIL progeny to exhibit expression of the gene. Over half (289/489) of the genes with segregation for the presence of transcripts exhibit this type of pattern. This pattern could be caused by a strong *cis-*regulatory variant or actual difference in genome content such as PAV [6], [59]. The mapping of regulatory variation for these 289 genes revealed that many of them can be attributed to variation mapping to the location of the gene itself and likely reflect sequence differences in regulatory regions or content variation. Alternatively, the presence/absence of a transcript could reflect a strong *trans-*regulatory variant and a subset of the genes do exhibit *trans-*eQTL. This set of genes with expression in one parent and roughly 50% of RILs are expected based on previous studies of maize genome content variation and regulatory variation [68].

Many of the genes with segregation for the presence of transcripts exhibit other, unexpected, patterns of expression. These include genes that are expressed in both parents but a few RILs, genes expressed in neither parent but many of the RILs and other patterns. These segregation patterns are not expected to result from traditional single, gene segregation. We did not find evidence that there was segregation for the presence/absence of these genes within the genomic DNA of progeny. It is quite possible that many of these unexpected patterns of segregation for transcript presence reflect epigenetic or small-RNA based regulatory mechanisms. For instance, an example from tomato illustrates that a miRNA present in one of the parents can become detectably expressed in all the hybrids and their progeny [47]. In addition, there are examples of molecular dominance in siRNA levels and DNA methylation in Arabidopsis F1 plants [69]–[70]. It will be important to further understand the mechanisms that generate these unexpected patterns of segregation to understand the inheritance of traits in RIL populations.

There is a growing appreciation for the qualitative variation among the genomes and transcriptomes of maize inbreds. Inbreds of maize can have substantial variation for gene content [6]–[7], [59], [71]. These inbreds can also have substantial variation for the presence of transcripts [29], [34]. The F1 genotypes will contain the full set of genes found in both parents and generally tend to express this full set leading to a potential contribution to heterosis [72]. In this study, we showed that the RILs can also vary in transcriptome content relative to the parental genotypes. This leads to questions about the functional consequences of variation in transcriptome content. Many of the studies on genome content and variation in transcriptome content have found that the variable genes are under-represented for syntenic genes with functional annotations. Consistently, we found that only 36 of the 210 genes with unexpected patterns of segregation for expression were located in syntenic chromosomal positions. The variation for the presence of expression for these genes may directly impact phenotypes. The other 174 genes include a number of inserted sequences relative to gene order in other grass species. The maize genome is known to be littered with gene fragments that have been captured and mobilized by transposons [14], [22]–[23], [73]. In many cases, the presence of these gene fragments is variable among maize genotypes [14]–[15], [74] and can contribute to novel transcripts [24]. Here we provide evidence that the presence/absence of transcripts from these gene fragments can act to modulate the expression level of the full-length parent gene. This suggests that some of the qualitative variation for gene fragment transcripts acts to provide a *trans-*acting regulator for the full-length gene and suggests a mechanism for the origin of selectable variation in expression level for single genes.

## Materials and Methods

### Plant materials

A maize IBM (Intermated B73×Mo17) RIL population derived from the cross of the inbred lines B73 and Mo17 [50] was used to assess segregation of gene expression. At least 10 seedlings per genotype of 105 IBM RILs and their parents were planted in a single growth chamber. A randomized block design was employed with three replicates. The order of the flats within each block was rotated daily to minimize the effects of local environmental variation. Fourteen days after planting, at least 6 healthy seedlings were harvested and a 4 mm cubic tissue including the shoot apex were dissected and pooled for each genotype-replication combination. After separately grinding tissue from each genotype-replication pool in liquid nitrogen, RNA was extracted using the TRIzol and Qiagen RNeasy mini kit following the manufacturer's instructions.

### RNA–seq and bioinformatic analysis

The three replicate RNA samples of each genotype were pooled with barcoding. RNA sequencing libraries were prepared and sequenced using the Illumina Hi-Seq2000 with 103–110 cycles. The resulting sequencing data were trimmed and aligned to the B73 reference genome v2 (AGPv2) [2] by Data2Bio (http://www.data2bio.com/). The majority (69–80%) of the trimmed reads were uniquely mapped and 94% of mapped reads were located in annotated gene regions. The uniquely-mapped reads were further analyzed for SNPs and read counts per genes in the RILs and their parents. RPKM values were determined using Cufflinks v0.9.3 (http://cufflinks.cbcb.umd.edu/) based on the uniquely mapped reads of each genotype. The AGP v2 5b maize genome annotation was used as a reference, while maximum intron length = 60,000 bp and the quartile normalization option were employed. To establish a threshold for detectable expression, we conducted global permutation tests with 10,000 randomly selected non-genic fragments from B73 RNA-seq data [75]. We found the RPKMs were 0.055, 1.03, 2.02 and 5.41 as cutoffs for gene expression at different significant levels of FDR = 0.05, 0.01, 0.005 and 0.001, respectively. For the initial analyses, a transcript presence/absence was assessed using a threshold of 0.055 RPKM. For the more stringent analysis of unexpected segregation patterns a threshold of 1.03 RPKM was employed and gene presence required values >1.03 and absence required a value of 0.0. Intermediate values were not assigned presence or absence calls.

### Global expression analysis

The 22,242 genes expressed in more than 90% of the RILs and the parents were used to interrogate the global expression variation. The population mean and coefficient of variation of gene expression levels were summarized for the attributes of the RIL population, whereas the absolute value of log2 of the expression-level in B73 divided by the level in Mo17 was used for the expression fold change between B73 and Mo17. The Kolmogorov-Smirnov test was applied to judge whether the expression levels of genes fit a normal distribution in the RIL population. The τ statistic, introduced by Bessarabova et al. [52], was employed to distinguish between one-modal (normal) and bimodal distributions. We simulated 10,000 normal distribution data (μ = 0, σ = 1), each containing 105 numbers, to obtain the global threshold of τ = 3.24 (*P* = 0.01). We treated the expression levels, which did not fit either normal or bimodal distribution, as unclassified distribution. The relationship between coefficient of variation and abs(log2(B73/Mo17)), and the relationship between τ value and abs(log2(B73/Mo17)) of the variation of global gene expression were assessed by Pearson's product-moment correlation analysis in R (http://www.R-project.org).

Ten randomly selected genes with expression-level (RPKM) ranging from 0.05 to 2552.91 were selected to validate the expression profiling accuracy of RNA-seq by quantitative RT-PCR (qRT-PCR) using the same RNA samples as the ones used for RNA-seq. For qRT-PCR, cDNA samples ware amplified using the iQ SYBR Green Supermix on the CFX96 Real-Time PCR detection system (Bio-Rad, Hercules, CA). Each PCR reaction contained 25 µl of reagent, consisting of 5 µl cDNA; 12.5 µl of the iQ SYBR Green Supermix; 2.5 µl of nuclease-free water; and 5 µl of the forward and reverse primers (1 µM stock). The qRT-PCR conditions included an initial incubation at 95°C for 3 min, followed by 40 cycles of 95°C for 10 sec, 58°C for 20 sec, and 72°C for 25 sec.

To test the expression pattern of the paramutation-like genes, we examined gene expression in the shoot apex from 18 individuals from an F2 population derived from a cross between B73 and Mo17. The F2 individuals, Mo17 and B73 were grown in a growth chamber using similar conditions as those used to obtain the RNA-seq data from the RIL population. RNA samples from the shoot apex were isolated from 2-week old seedlings and reverse-transcribed into the first strand cDNAs for the qRT-PCR quantification. Ten randomly selected paramutation-like genes were examined for the relative quantitation of expression level in the F2 individuals and their parents. qRT-PCR was performed with the SYBR Green master mix according to the manufacturer's instructions (Applied Biosystems, Carlsbad, California). Three replicates were conducted to calculate the average and standard deviation of expression levels. The 2^−ΔΔCT^ method was employed to calculate the relative quantitation of expression levels with the housekeeping gene *Actin* as the endogenous control and B73 as the reference genotype.

To validate the unexpected expression patterns we conducted two experiments. In the first experiment, we replanted 10 IBM RIL genotypes, using the same growth conditions as used in the RNA-seq experiment, with 10 plants per genotype and sampled the shoot apices of the seedlings 14 days after planting. RNA was isolated from at least 6 healthy plants per genotype. In the second experiment, we tested the expression variation of genes with unexpected expression patterns in 18 individuals from an F2 population derived from a cross between B73 and Mo17. A total of 55 genes with unexpected expression patterns were randomly selected for validation. RT-PCR was conducted using a Touchdown PCR program [76]. Two cycling phrases were set for the Touchdown PCR program: the TM reduced from 72°C to 62°C by 1°C every successive cycle in the first phrase with 10 cycles, while 25 other cycles were used for the amplification in the second phrase with TM = 62°C. Thus, 35 cycles were conducted. We also conducted genomic DNA PCR amplifications on the same RILs with the Touchdown PCR program on 8 randomly selected genes with unexpected expression patterns to check whether the extraordinary expression occurred only at the transcript level. The concentration of the template cDNA and DNA was 10 ng/µl for all the validations of RT-PCR and genomic PCR. All primer information can be found in Table S12.

To examine the expression patterns in hybrids of B73 and Mo17 for the paramutation-like genes, we dissected shoot apices from 10 plants from B73, Mo17 and their reciprocal hybrids, isolated RNA and conducted RNA-seq. For this experiment, the plants were grown in the same growth chamber conditions used for the original RNA-seq experiment, using consistent protocols for sampling, library preparation, RNA-seq and analysis.

For the analyses of attributes of genes with unexpected presence/absence expression patterns, we downloaded the gene family information of the whole B73 gene set from EnsemblPlants (http://plants.ensembl.org/index.html). Gene family relationships were constructed through EnsemblCompara GeneTrees by using the phylogenetic approach [77]. The syntenic information of maize genes was obtained from the CoGe database (http://genomevolution.org/CoGe/).

### Transposon enrichment effect analyses

We annotated 20 Kb of flanking sequence for the genes with unexpected expression patterns (Type I, Type II and Type IIIA and Type IIIB) in 5 Kb windows as a fragment Bin by RepeatMasker (http://repeatmasker.org). As controls, 210 genes were randomly selected and 10,000 permutations were conducted. Then, we annotated the adjacent fragments from 5 Kb upstream and downstream for all the FGS and summarized the number of all the different kinds of transposon-like sequences in the adjacent fragment of genes.

### eQTL mapping

Data2Bio (http://www.data2bio.com/) identified 648,230 putative SNPs in 28,603 genes (72% of all maize genes) using RNA-Seq reads from the RILs and their parents. High quality unique SNP markers with minimal missing data in the RILs were selected, grouped and integrated into chromosomes before constructing the genetic map. Maximum Likelihood Estimation with minimal threshold LOD score = 3.0 by JoinMap 4.0 [78] was employed to construct a high-resolution genetic map. The expression-levels of 22,242 genes were treated as expression traits (e-traits) for the global gene eQTL mapping. The genetic determinants controlling variation in e-traits were mapped via composite interval mapping [79]–[80] with a walking speed of 1 cM in the procedure of SRmapqtl and Zmapqtl of QTL cartographer [81]. A global permutation with 1000 randomly selected e-traits×1000 replicates were done as a representative null distribution of 1,000,000 maximum likelihood ratio test (LRT) statistics. A global permutation threshold as the significant cutoff of eQTL mapping was obtained at a significance level of 0.05, giving a likelihood ratio test value of 19.23, which corresponds to a Logarithm of Odds (LOD) score of 4.17. The range with a 1.0 LOD drop on each side from the LOD peak point was selected as the confidence interval. If two adjacent peaks overlap in less than 10 cM, we treated them as one eQTL. A global permutation of randomly distribution of *trans-*eQTLs along the whole maize genome was performed to find the threshold of *trans-*eQTLs hotspots. One thousand of the maximum number of *trans-*eQTL scattering in 1 Mb genomic region of each permutation were obtained to compute the cutoff of hotspots. Further, we took gene density into account to rule out the gene number factor for the identification of *trans-*eQTL hotspots. For global *trans-*eQTLs hotspots, the cutoff (#_*trans-*eQTLs/(Mb×#_genes)) was 1.25. The GO enrichments and the pathway enrichments of the regulated genes by hotspots were conducted using BiNGO plugin [82] in Cytoscape [83] based on the annotation information from AgriGO [84] and MaizeCyc database [58], respectively.

### Epistasis scan of the transcriptomic variations of genes with unexpected expression patterns

The epistasis scan with all possible pairwise marker interactions for the genes with unexpected expression patterns was conducted with a generalized linear model. We employed an α-level of 0.05 (*P*<2.1E-06), which was adjusted by following the suggestion of dividing the α-level by the number of possible independent pairwise interactions among recombinant blocks [85].

### Relating CNVs to transcriptome variation

We obtained genomic variation information between B73 and Mo17 from Springer et al. 2009. The formula of CGH signal abundance of B73 and Mo17 of log2(Mo17/B73) were used to classify different CGH types [59]. The segments with a peak at log2(Mo17/B73) = 0 were simply classified as B = M, while the segments with a peak at log2(Mo17/B73) = 20.43 were classified as B73<Mo17_SNP. B = M_int represents segments with an intermediate value between 0 and 20.43. Mo17>B73_CNV shows segments that are predicted to occur in more copies in Mo17 than in B73. B>M_CNV indicates segments containing significantly more copies in B73 than in Mo17, while B>M_int represents segments having intermediate more copies in B73 than in Mo17. B>M_PAV shows segments present in B73 but absent in Mo17. Of these genomic variants we mainly focused on CGH segments B>M_int, B>M_CNV, B>M_PAV and M>B_CNV for the relationship analyses between genomic variation and transcriptome variation in the maize IBM RIL population. First, we coordinated genes with CGH segments by coding scripts to compare the coordinates of genes (according to the annotation of the maize reference genome AGPv2) with the CGH segments. Four main relationships could be obtained as genes entirely within CGH segments, genes intersecting CGH segments, genes in regions having multiple CGH segments, and other. Second, we filtered expressed genes and CGH segments. We limited the analysis to the expressed genes, which we defined as those displaying a normalized expression value (RPKM) of at least 1.03 (corresponding to 21 reads per gene, FDR = 0.01) in more than 40% of the samples. Further, we considered the pair-wise datasets between genes and CNVs only if genes were expressed in at least 40 samples for each inferred genotype (B73 and Mo17) in the RIL population. Finally, we conducted eQTL mapping of genes with CNVs nearby, for the inference of associations between structural variation and expression levels.

### RNA–seq data availability

The raw RNA-seq data on shoot apices of the IBM RIL population used in this study were submitted to NCBI's Sequence Read Archive (SRA) with accession number SRA055066 and will be released to public after approval of publication. The transcriptome profiling data were also deposited in MaizeGDB (http://www.maizegdb.org/).

## Supporting Information

Figure S1 Expression level correlation between RNA-seq and qRT-PCR. The x-axis denotes the RPKM value quantified by RNA-seq, while the y-axis shows the average CT value obtained via qRT-PCR. The validations were done on ten randomly selected genes that exhibit a range of mean-expression levels in seven RILs and the two parents. The *r* in the graphs indicates the correlation coefficient. The graphs (A)–(I) represent the genes: GRMZM2G005040, GRMZM2G149452, AC206951.3_FG017, AC199782.5_FG001, AC207890.3_FG002, AC199782.5_FG002, AC206642.4_FG001, GRMZM2G108348, and GRMZM2G152908, respectively. ** represents the significant level (P<0.10). Seven genes exhibited significant correlation coefficients between the RPKM derived from the RNA-seq data and the average cycle threshold (CT) value derived from the qRT-PCR data. Two genes in D (AC199782.5_FG001) and G (AC206642.4_FG001) did not exhibit significant correlation between the RNA-seq and qRT-PCR results. However, these two genes have very little variation in expression among the RILs and therefore we might not expect a strong correlation of variance between the two technologies. The remaining gene (GRMZM2G044856), which exhibited the lowest RPKM value, could not be detected by qRT-PCR.
(TIF)

Figure S2 Distribution of expression levels for all genes with paramutation-like expression patterns. The y-axis shows the RKPM value for the normalized expression levels. The x-axis represents all genes with paramutation-like expression patterns. The blue triangle represents B73, while the red diamond indicates Mo17. All genes with paramutation-like expression patterns were expressed in the RILs at the expression levels close to one of the parents. The majority of these genes (124/145) had patterns in which the RILs were all expressed at levels similar to the lower parent, while a few genes (21) were expressed at levels close to the higher parent.
(TIF)

Figure S3 Distribution of d/a values for all differentially expressed genes (2-fold changes) and genes with a paramutation-like pattern. (A) Distributions of d/a ratios in the hybrids and the two parents for paramutation-like genes with lower parental expression level in the RILs. (B) Distributions of d/a ratios in the hybrid and the two parents for paramutation-like genes with higher parental expression level in the RILs. The d/a values represented here indicate the hybrid expression levels relative to the low-parent and high-parent levels. In total, 63 of these paramutation-like genes showed dominant expression patterns in the hybrids (B73×Mo17 and Mo17×B73), in which the genes were expressed at the levels close to one of the parents but significantly different (*P*<0.05) from the other parent.
(TIF)

Figure S4 Distribution of expression levels in F2 individuals and RILs for ten genes with paramutation-like expression patterns. For each gene shown along the x-axis, the two y-axes show the expression level for the relative quantitation values in the F2 individuals by qRT-PCR and the RPKM value for the normalized expression level by RNA-seq in the RILs. The blue triangle represents B73, while the red diamond indicates Mo17. (A) Seven (*GRMZM2G015818*, *GRMZM2G044132*, *GRMZM2G137696*, *GRMZM2G453805*, *GRMZM2G066049*, *GRMZM2G089493* and *GRMZM2G349791*) of the 10 paramutation-like genes (70%) exhibited similar expression patterns in the F2 individuals as observed in the RILs. (B) Three genes (*GRMZM2G031331*, *GRMZM2G084958* and *GRMZM2G102356*) did not exhibit paramutation-like expression patterns in the F2 individuals.
(TIF)

Figure S5 Characteristics of *cis-*eQTL and *trans-*eQTL. (A) Shows the *R* ^2^ frequency distribution of *cis-*eQTL and *trans-*eQTL. Green bars represent *trans-*eQTL, blue bars show *cis-*eQTL and red area is the overlap in the graph between *cis-*eQTL and *trans-*eQTL. The boxplot shows the *R* ^2^ comparison among *cis-*eQTLs, *trans-*eQTLs, and *trans-*eQTLs in *trans*-eQTL hotspots. In (B), (C) and (D), the x-axis is the absolute value of log2 of expression-level in B73 divided by the level in Mo17. (B) The relationship between the proportion of e-trait distribution and the parental difference. (C) The relationship between *R* ^2^ variation of *cis-*eQTLs and the parental difference. The y-axis in graph (B) shows the *R* ^2^ value of *cis-*eQTLs. (D) The relationship between *R* ^2^ variation of *trans-*eQTLs and the parental difference. The y-axis shows the *R* ^2^ value of *trans-*eQTLs.
(TIF)

Figure S6 RT-PCR validation of randomly-selected genes with unexpected expression patterns. RT-PCR was conducted for a set of genes with unexpected expression patterns using a subset (10) of the same RILs used for RNA-seq but grown in an independent experiment. All RT-PCR assays were conducted with Touch-Down PCR programs of 35 PCR amplification cycles. Graphs (A), (B), (C) and (D) show the validation of genes with Type I, Type II and Type IIIA and Type IIIB patterns, respectively. The type I pattern represents genes that were expressed in both parents but were not detected (RPKM = 0) in over 10% of the RILs. The type II pattern shows genes that were not detected (RPKM = 0) in the parents but were detected in at least 10% of the RILs. The type III patterns include genes that were expressed in one parent but not the other and had expression in very few RILs (type IIIA) or the majority of the RILs (type IIIB).
(TIF)

Figure S7 The genes with unexpected segregation for expression are present in the genomic DNA of all samples. PCR was performed on genomic DNA and RT-PCR was performed on RNA for a subset of genotypes for eight genes with unexpected expression patterns. All eight genes were detected in the genomic DNA of all samples but exhibit segregation for gene expression. All RT-PCR and genomic PCR assays were conducted using the Touch-Down PCR program with 35 cycles.
(TIF)

Figure S8 The expression levels and standard deviations of genes with unexpected expression patterns compared with those of all other expressed genes. The genes with unexpected expression patterns (UEP) exhibited the same expression levels and standard deviations as all (All) other expressed genes in the RILs.
(TIF)

Figure S9 Examples of genes with unexpected expression patterns controlled by two-locus interactions. The x-axis represents different types of genotypes of the RILs. *A* and *B* indicate two independent loci, *AABB* represents the Mo17 genotype, while *aabb* shows the B73 genotype. The y-axis indicates the normalized expression levels of the RILs and their parents. The blue triangle indicates the expression level in B73, while the red diamond indicates the expression level in Mo17. (A) and (B) show that these genes with expression in only ∼25% of the RILs could be explained by a two locus interaction, while (C) and (D) represent genes that exhibit expression in ∼75% of the RILs and could also be controlled by a two locus interaction. (A), (B), (C) and (D) represent multiple locus interactions for the expression patterns of Type II, Type IIIA, Type I and Type IIIB, respectively. Taken together, 91% of genes with expression in only ∼25% or 75% of the RILs were identified to be controlled by pair-wise locus interactions.
(TIF)

Figure S10 Schematic diagram of the proportion of genes with different transposons in the flanking genomic regions. The x-axis represents different transposons, while the y-axis shows different flanking genomic blocks (5 Kb/block), of which the minus (−) and plus (+) indicate the upstream from the transcriptional start site of the gene and the downstream region from the transcriptional terminal site of the gene, respectively. “UEP” represents the genes with unexpected expression patterns, whereas “Control” shows the randomly-selected genes from the filtered-evidence gene set [2].
(TIF)

Table S1 Summary of RNA-seq data derived from shoot apices of 105 IBM RILs and B73 and Mo17. The preliminary RNA-seq analyses (RNA-seq mapping and population SNP calling) were conducted by Data2Bio (http://www.data2bio.com/) by mapping trimmed reads to the B73 reference genome AGPv2 (www.maizesequence.org).
(XLS)

Table S2 Paramutation-like genes detected in the maize IBM RIL population. ^a^ represents the number of standard deviations of difference between B73 and the RIL population, ^b^ represents the number of standard deviations between Mo17 and the population mean. The expression levels in the RILs and their parents were normalized by RPKM. ^c^ shows the standard deviation of expression levels in the RIL population.
(XLS)

Table S3 eQTL mapping of the maize shoot apex. ^a^, ^b^ indicate the chromosome and genetic position of e-traits, respectively; ^c^ shows the physical chromosomal location on the B73 reference genome (AGPv2) of e-traits; ^d^ shows the middle physical position (equals the sum of the position of the transcription start site and the termination site divided by 2) of e-traits; ^e^ indicates the genetic position of the peak of the eQTL; ^f^ is the genetic position of the inferior support interval left bound of the eQTL; ^g^ is the genetic position of the inferior support interval right bound of the eQTL; ^h^ represents the physical position of the peak of the eQTL on the B73 reference genome (AGPv2); ^i^ is the Logarithm of Odds (LOD) score of the eQTL; ^j^ is the additive effect, the positive value indicates that the allele from Mo17 increases the phenotypic value; ^k^ indicates the amount of expression variation of the e-trait explained by the eQTL; Type shows the relationship between e-traits and the eQTLs.
(XLS)

Table S4 Summary of *trans-*eQTL hotspots. ^a^, ^b^ show the number of *cis-* and *trans-*eQTLs in each eQTL hotspot, respectively; ^c^ indicates the number of eQTLs, where the B73 allele increased the expression level; ^d^ indicates the number of eQTLs, where the Mo17 allele increased the transcript-level in the RIL population. ^e^shows the significant level deviating from the random distribution between B73 and Mo17.
(XLS)

Table S5 GO annotation of regulated genes at each *trans-*eQTL hotspot. The GO enrichment analysis of the regulated genes at each *trans-*eQTL hotspot was conducted using BiNGO plugin in Cytoscape based on the annotation information from AgriGO.
(XLS)

Table S6 MaizeCyc enrichment of regulated genes at each *trans-*eQTL hotspot with at least 200 targets. The pathway enrichment of the regulated genes by hotspots was conducted using BiNGO plugin in Cytoscape based on the annotation information from MaizeCyc database.
(XLS)

Table S7 The number of expressed genes intersecting with CNV/PAV. Mo17>B73_CNV shows segments that are predicted to occur in more copies in Mo17 than in B73. B>M_CNV indicates segments containing significantly more copies in B73 than in Mo17, while B>M_int represents segments having intermediate more copies in B73 than in Mo17. B>M_PAV shows segments present in B73 but absent in Mo17.
(XLS)

Table S8 Expression of genes entirely within CNV/PAV regions. ^a^ indicates the number of genes with expression level of B73 significantly higher than Mo17; ^b^ represents the number of genes with expression level of B73 significantly lower than Mo17; ^c^ shows the number of genes with no expression changes between B73 and Mo17. Mo17>B73_CNV shows segments that are predicted to occur in more copies in Mo17 than in B73. B>M_CNV indicates segments containing significantly more copies in B73 than in Mo17, while B>M_int represents segments having intermediate more copies in B73 than in Mo17. B>M_PAV shows segments present in B73 but absent in Mo17.
(XLS)

Table S9 Genes with unexpected expression patterns. ^a^ shows the number of RILs in which no read of the target gene was detected. ^b^ the number of RILs in which the target gene was expressed. ^c^ segregation rate was tested by using Chi-square test. ^d^ Genetic model was inferred according to the segregation rate. ^e^ I, U and S in column “Syntenic Code” represent the syntenic relationships among grass species: inserted, unknown and syntenic, respectively. ^f^ the classification of whole genome duplication.
(XLS)

Table S10 Expression correlation between genes with unexpected patterns and their homologous genes. ^a^ shows the middle physical position (equals the sum of the position of the transcriptional start site and the terminal site divided by 2) of the gene; ^b^ is the genetic position in the IBM population; ^c^ indicates the coefficient of correlation; ^d^ is the action type, cis indicates the two duplicate genes are located in the same genomic region, while trans shows the two duplicate genes are not in the same genomic region. In the column of “Syntenic Classification”, S, I, and U represent Syntenic, Inserted and Unknown, respectively.
(XLS)

Table S11 Co-regulated genes at hotspots and genes with cis-eQTL near trans-eQTL hotspots. ^a^ refers to trans-eQTL hotspts. ^b^ is the gene with *cis-*eQTL underneath the hotspot; ^c^ indicates the number of regulated genes by hotspot; ^d^ is the number of shared genes co-expressed with a *cis-*regulated gene and also found within a hotspot.
(XLS)

Table S12 Primer information used for validation. The “partial” in Validation Status column means the expression in a few RILs (less than 2 RILs out of 10 tested RILs) of the other independent replication did not match with RNA-seq but the gene still showed an unexpected expression pattern.
(XLS)
