# Correction: Mendelian and Non-Mendelian Regulation of Gene Expression in Maize

**DOI:** 10.1371/journal.pgen.1007234

**Published:** 2018-02-14

**Authors:** Lin Li, Katherine Petsch, Rena Shimizu, Sanzhen Liu, Wayne Wenzhong Xu, Kai Ying, Jianming Yu, Michael J. Scanlon, Patrick S. Schnable, Marja C. P. Timmermans, Nathan M. Springer, Gary J. Muehlbauer

There are errors with the eQTL effects reported in this article. The effects should be reversed; specifically, the base allele in the contrast to derive the additive effects should be B73, rather than Mo17. The authors apologize for the errors and provide further details and corrections to the text, the Fig 3 legend, and Tables 1, S3 and S4 below. The reversal of the eQTL effect does not affect the main conclusions of the manuscript, as subsequent results were gained by using the RPKM values from individual RILs and not the eQTL mapping results.

In Table 1, the values of the 8^th^ and 9^th^ columns (B73^c^ and Mo17^d^) should be switched. Additionally, instead of ten eQTL hotspots that contain at least 200 eQTL there are only nine eQTL hotspots that contain at least 200 eQTLs. The corrected version of Table 1 is below, and includes only nine hotspots with at least 200 eQTLs.

**Table 1:** *Trans*-eQTL hotspots with at least 200 *trans*-eQTLs.

Hotspot_name	Chr	StartPos (Mb)	EndPos (Mb)	#_*cis*^a^	#_*trans*^b^	#_eQTL/ (Mb×#_gene)	B73^C^	Mo17^d^	Sig.Bias^e^	GO Term enrichment	MaizeCyc enrichment
Zm_eQTL_HS14	2	3	5	56	353	3.18	64	289	4.77E-33	Yes	No
Zm_eQTL_HS20	2	202	206	70	263	2.10	102	161	2.75E-04	Yes	No
Zm_eQTL_HS25	3	4	6	28	228	3.51	118	110	5.96E-01	Yes	No
Zm_eQTL_HS29	3	214	218	63	336	2.95	249	87	9.76E-19	No	No
Zm_eQTL_HS35	4	157	160	30	379	5.92	58	321	1.38E-41	Yes	Yes
Zm_eQTL_HS37	4	176	182	45	420	2.80	146	274	4.22E-10	Yes	Yes
Zm_eQTL_HS41	4	236	238	38	259	2.78	17	242	2.04E-44	Yes	Yes
Zm_eQTL_HS65	7	156	160	51	274	2.14	192	82	3.03E-11	Yes	Yes
Zm_eQTL_HS95	10	145	147	35	221	2.83	157	64	3.95E-10	Yes	Yes

^a,b^: Indicates the number of *cis*- and *trans*-eQTLs in each eQTL hotspot, respectively.

^c^: Indicates the number of eQTLs, where the B73 allele increased the expression level in the RIL population.

^d^: Indicates the number of eQTLs, where the Mo17 allele increased the expression level in the RIL population.

^e^: Shows the significance level deviating from the random distribution between B73 and Mo17. The GO enrichments and the pathway enrichments of the regulated genes by hotspots were conducted using BiNGO plugin in Cytoscape based on the annotation information from AgriGO and MaizeCyc database, respectively. The results of GO enrichments and pathway enrichments are in Table S5 and Table S6, respectively.

In the legend for Fig 3, the phrase “the additive effects of the *trans*-eQTLs of **Mo17** alleles” should be “the additive effects of the *trans*-eQTLs of ***B73*** alleles” and the phrase “***10*** *trans*-eQTLs hotspots” should be “***nine*** *trans*-eQTLs hotspots”. The corrected legend and a copy of the figure are included below.

**Fig 3:**
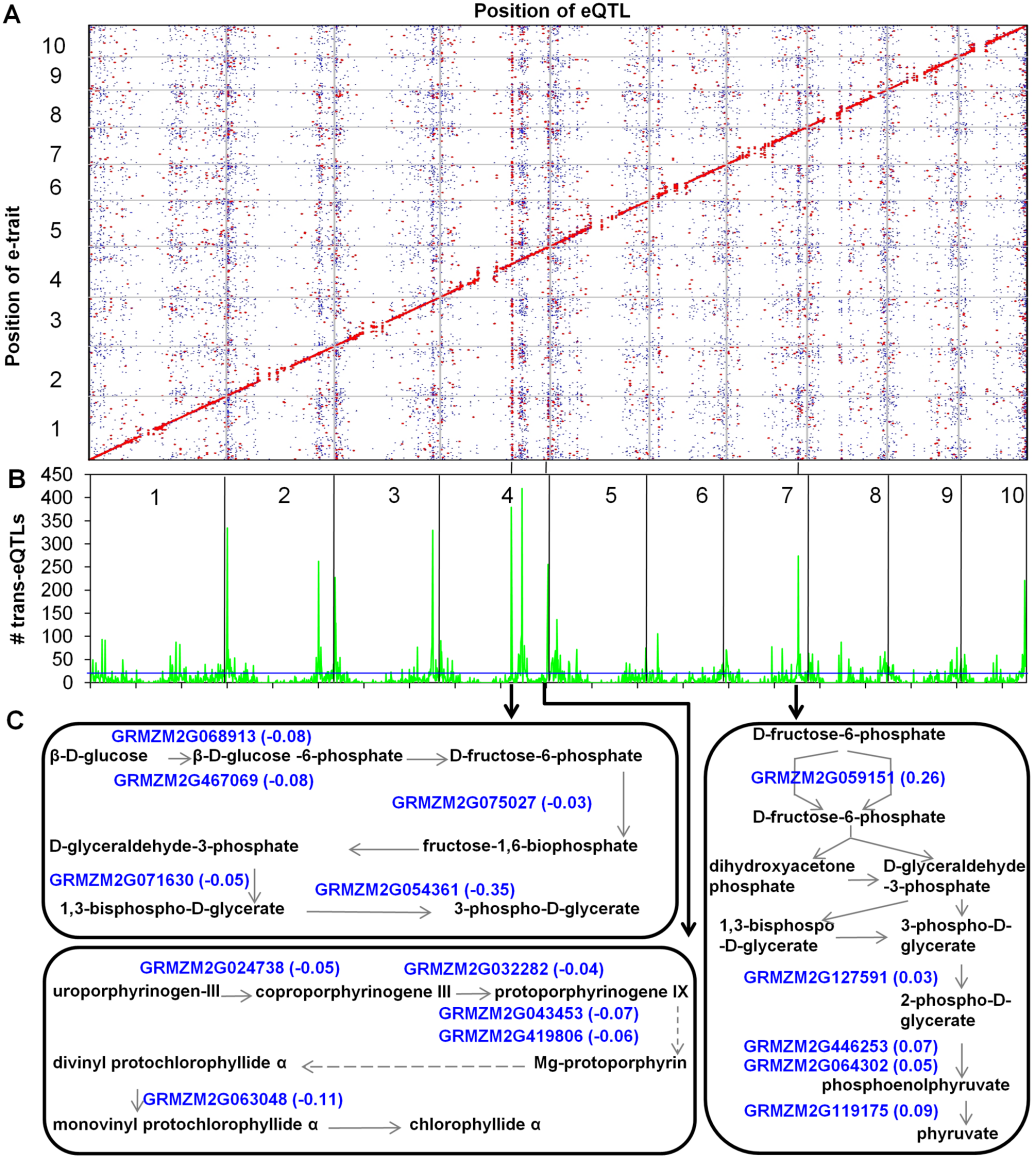
eQTL mapping, *trans*-eQTL hotspots, and pathways regulated by three *trans*-eQTL hotspots. (A) Genomic distribution of eQTLs identified in maize shoot apices. The x-axis indicates the genomic positions of eQTLs, while the y-axis shows the genomic positions of expressed genes (e-traits). The 10 maize chromosomes are separated by grey lines. The color of each point reflects the *R*^2^ value. eQTLs with *R*^2^ values greater than 20% were plotted in red, *R*^2^ values less than 20% are indicated in blue. Totally, 30,774 eQTLs were divided into 11,504 (∼37%) *cis*-eQTLs and 19,270 (∼63%) *trans*-eQTLs. (B) The distribution of *trans*-eQTLs hotspots. The x-axis shows the genomic position of detected eQTLs (unit = 1 Mb), while the y-axis represents the number of *trans*-eQTLs in each 1 Mb length genomic region. The horizontal blue line for eQTL hotspots indicates the threshold, which is represented by the maximum number of *trans*-eQTLs expected to randomly fall into any interval with a genome-wide P = 0.01. The 10 maize chromosomes were divided by vertical black lines. The black lines linking (A) and (B) show several examples of the corresponding *trans*-eQTL hotspots in (A) and (B). A total of 96 *trans*-eQTLs hotspots were identified and nine *trans*-eQTLs hotspots regulated at least 200 *trans*-eQTLs. (C) Genes regulated by three *trans*-eQTL hotspots are involved in specific metabolic pathways. The expression levels of these genes in pathways were regulated by *trans*-eQTLs located in these hotspots. The numbers beside these genes are the proportional changes which were the additive effects of the *trans*-eQTLs of B73 alleles divided by the population mean of expression levels of the target genes.

In the Results section, the second paragraph under the sub-heading ‘Mapping the basis of expression level variation’ should be corrected to reflect the changes made to Table 1 and the Fig 3 legend:

“The genomic distribution of *trans*-eQTL was assessed in an attempt to identify potential *trans*-eQTL hotspots that might reflect substantial regulatory differences between B73 and Mo17. The analysis of *trans*-eQTL density in a 1 Mb (which is slightly larger than the average physical distance between adjacent markers with a recombination event) sliding window revealed 96 significant (*P*<0.01) *trans*-eQTL hotspots (Fig 3B and S4 Table), including nine major hotspots that contain at least 200 *trans*-eQTLs (Table 1). These hotspots have many more *trans*-eQTL than other genomic regions and in the majority (78%) of examples the target genes regulated at the *trans*-eQTL hotspots show a consistent pattern with significantly more target genes altered in expression in the same direction by the haplotype at the *trans*-eQTL hotspot (haplotype bias). More examples in which the Mo17 allele (49) at the *trans*-eQTL hotspot promoted higher expression of the target loci than the B73 allele (26) were identified. The lists of target genes regulated by each of the *trans*-eQTL hotspots were used to search for GO enrichments; 43% of the *trans*-eQTL hotspots target lists exhibited enrichments for at least one GO term (Table S5). We performed further analyses for the nine *trans*-eQTL hotspots that had at least 200 targets (Table 1). Eight of these nine *trans*-eQTL hotspots showed consistent haplotype bias (five for Mo17 and three for B73) and the targets for each of these hotspots had GO enrichments for at least one term. Multiple genes in the same MaizeCyc pathway [58] are observed to be co-regulated by the same *trans*-eQTL hotspot (Fig 3C, Table S6). These *trans*-eQTL hotspots may be due to functional differences in transcriptional regulators. At least in some cases it might be expected that differential expression of a regulator present at the *trans*-eQTL hotspot is the cause of the differences in *trans*-regulation.”

In S3 Table, the footnote is incorrect. The phrase “the allele from ***Mo17*** increases the phenotypic value” should read “the allele from ***B73*** increases the phenotypic value”. In S4 Table, the values in the 9^th^ and 10^th^ (B73^c^ and Mo17^d^) should be switched. Corrected versions of S3 and S4 Tables are provided below.

## Supporting information

S3 Table eQTL mapping of the maize shoot apex. ^a^, ^b^ indicate the chromosome and genetic position of e-traits, respectively; ^c^ shows the physical chromosomal location on the B73 reference genome (AGPv2) of e-traits; ^d^ shows the middle physical position (equals the sum of the position of the transcription start site and the termination site divided by 2) of e-traits; ^e^ indicates the genetic position of the peak of the eQTL; ^f^ is the genetic position of the inferior support interval left bound of the eQTL; ^g^ is the genetic position of the inferior support interval right bound of the eQTL; ^h^ represents the physical position of the peak of the eQTL on the B73 reference genome (AGPv2); ^i^ is the Logarithm of Odds (LOD) score of the eQTL; ^j^ is the additive effect, the positive value indicates that the allele from B73 increases the phenotypic value; ^k^ indicates the amount of expression variation of the e-trait explained by the eQTL; Type shows the relationship between e-traits and the eQTLs.
(XLS)

S4 Table Summary of *trans*-eQTL hotspots. ^a^, ^b^ show the number of *cis*- and *trans*-eQTLs in each eQTL hotspot, respectively; ^c^ indicates the number of eQTLs, where the B73 allele increased the expression level; ^d^ indicates the number of eQTLs, where the Mo17 allele increased the transcript-level in the RIL population. ^e^ shows the significant level deviating from the random distribution between B73 and Mo17.
(XLS)
