# Supplementary figures and images for: Mendelian and Non-Mendelian Regulation of Gene Expression in Maize
Source: PLoS Genet. 2013 Jan 17;9(1):e1003202. doi: 10.1371/journal.pgen.1003202 (PMC3547793; doi:10.1371/journal.pgen.1003202)

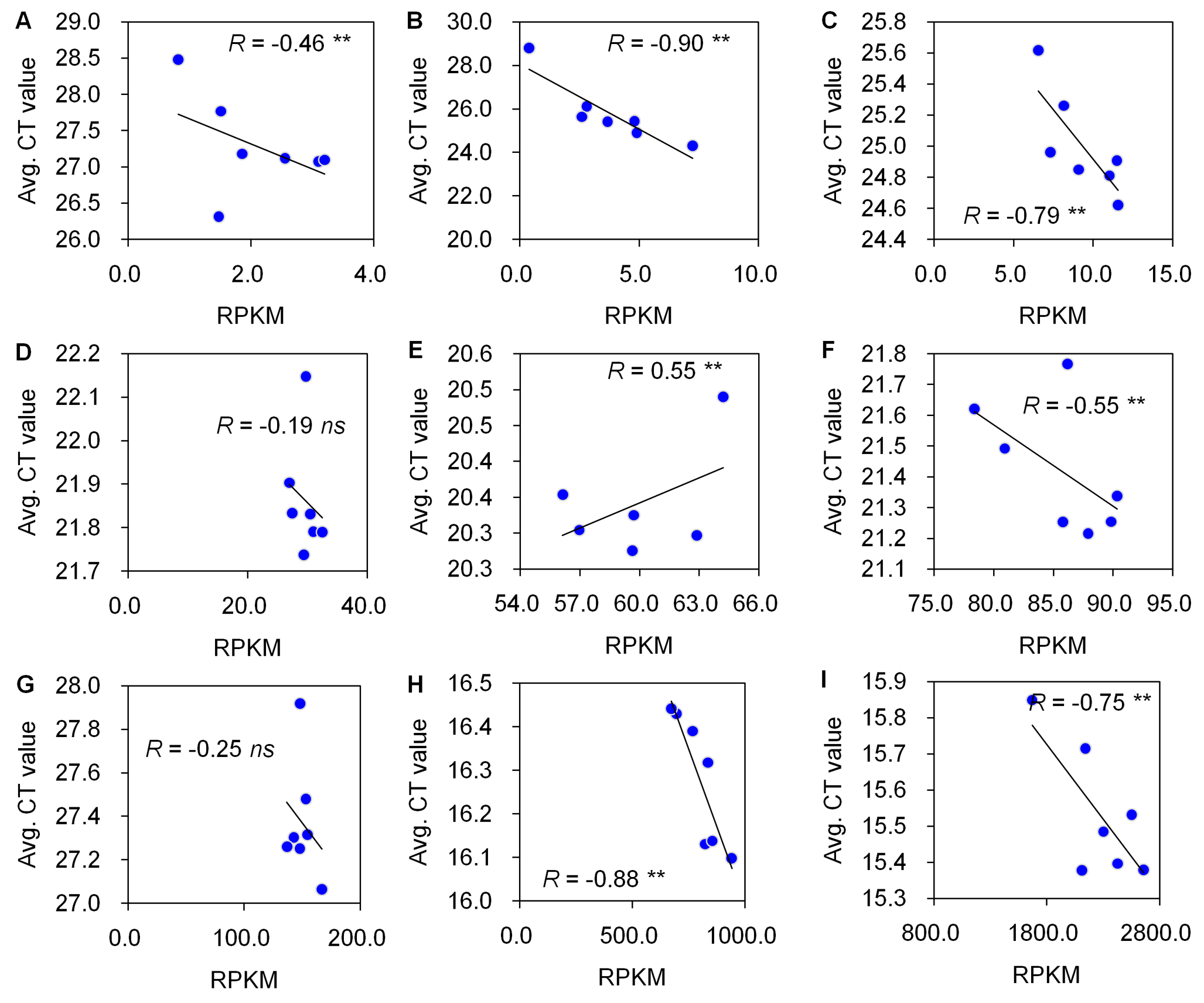

Supplement: Figure S1 — Expression level correlation between RNA-seq and qRT-PCR. The x-axis denotes the RPKM value quantified by RNA-seq, while the y-axis shows the average CT value obtained via qRT-PCR. The validations were done on ten randomly selected genes that exhibit a range of mean-expression levels in seven RILs and the two parents. The r in the graphs indicates the correlation coefficient. The graphs (A)–(I) represent the genes: GRMZM2G005040, GRMZM2G149452, AC206951.3_FG017, AC199782.5_FG001, AC207890.3_FG002, AC199782.5_FG002, AC206642.4_FG001, GRMZM2G108348, and GRMZM2G152908, respectively. ** represents the significant level (P<0.10). Seven genes exhibited significant correlation coefficients between the RPKM derived from the RNA-seq data and the average cycle threshold (CT) value derived from the qRT-PCR data. Two genes in D (AC199782.5_FG001) and G (AC206642.4_FG001) did not exhibit significant correlation between the RNA-seq and qRT-PCR results. However, these two genes have very little variation in expression among the RILs and therefore we might not expect a strong correlation of variance between the two technologies. The remaining gene (GRMZM2G044856), which exhibited the lowest RPKM value, could not be detected by qRT-PCR. (TIF) [file pgen.1003202.s001.tif]

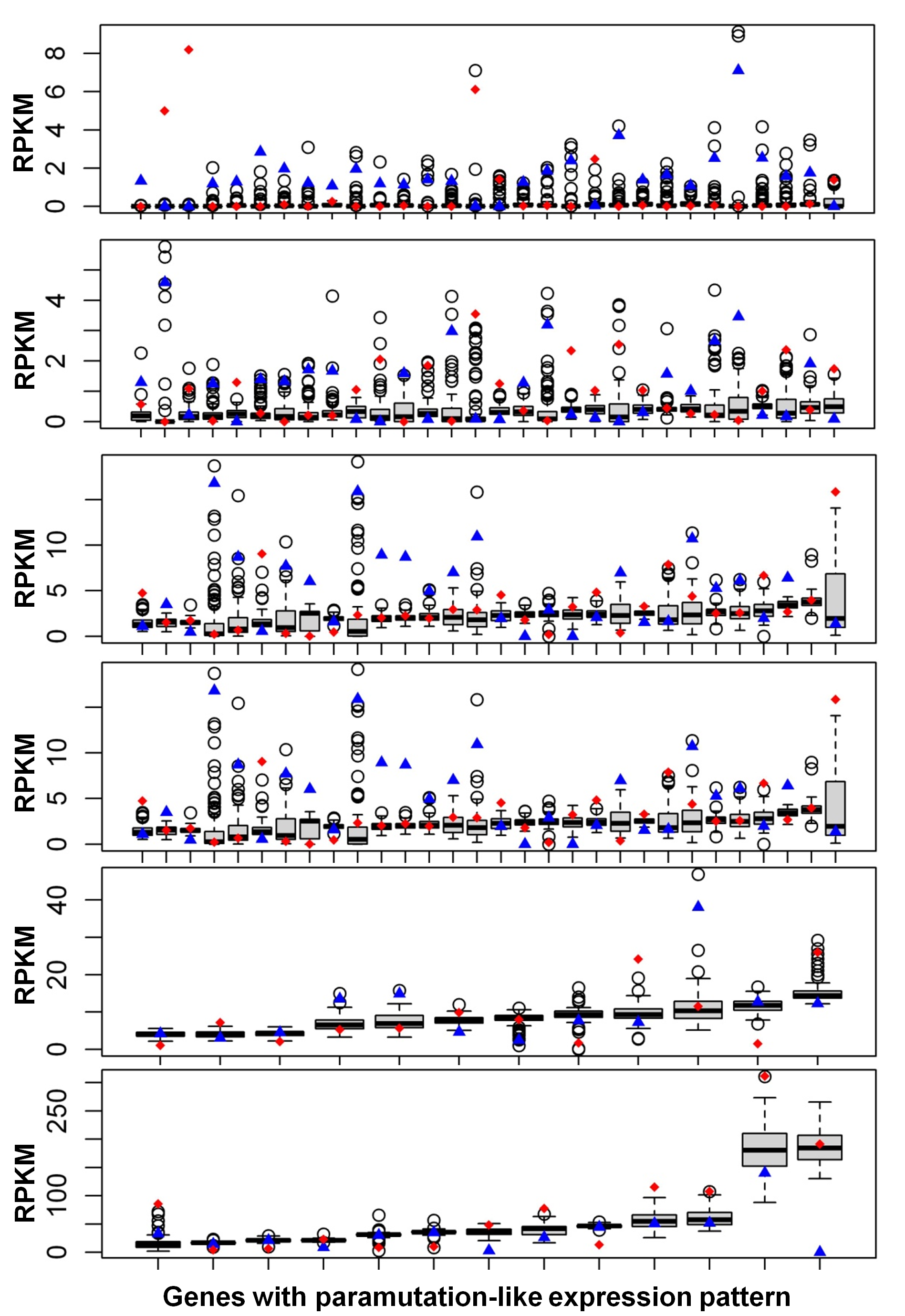

Supplement: Figure S2 — Distribution of expression levels for all genes with paramutation-like expression patterns. The y-axis shows the RKPM value for the normalized expression levels. The x-axis represents all genes with paramutation-like expression patterns. The blue triangle represents B73, while the red diamond indicates Mo17. All genes with paramutation-like expression patterns were expressed in the RILs at the expression levels close to one of the parents. The majority of these genes (124/145) had patterns in which the RILs were all expressed at levels similar to the lower parent, while a few genes (21) were expressed at levels close to the higher parent. (TIF) [file pgen.1003202.s002.tif]

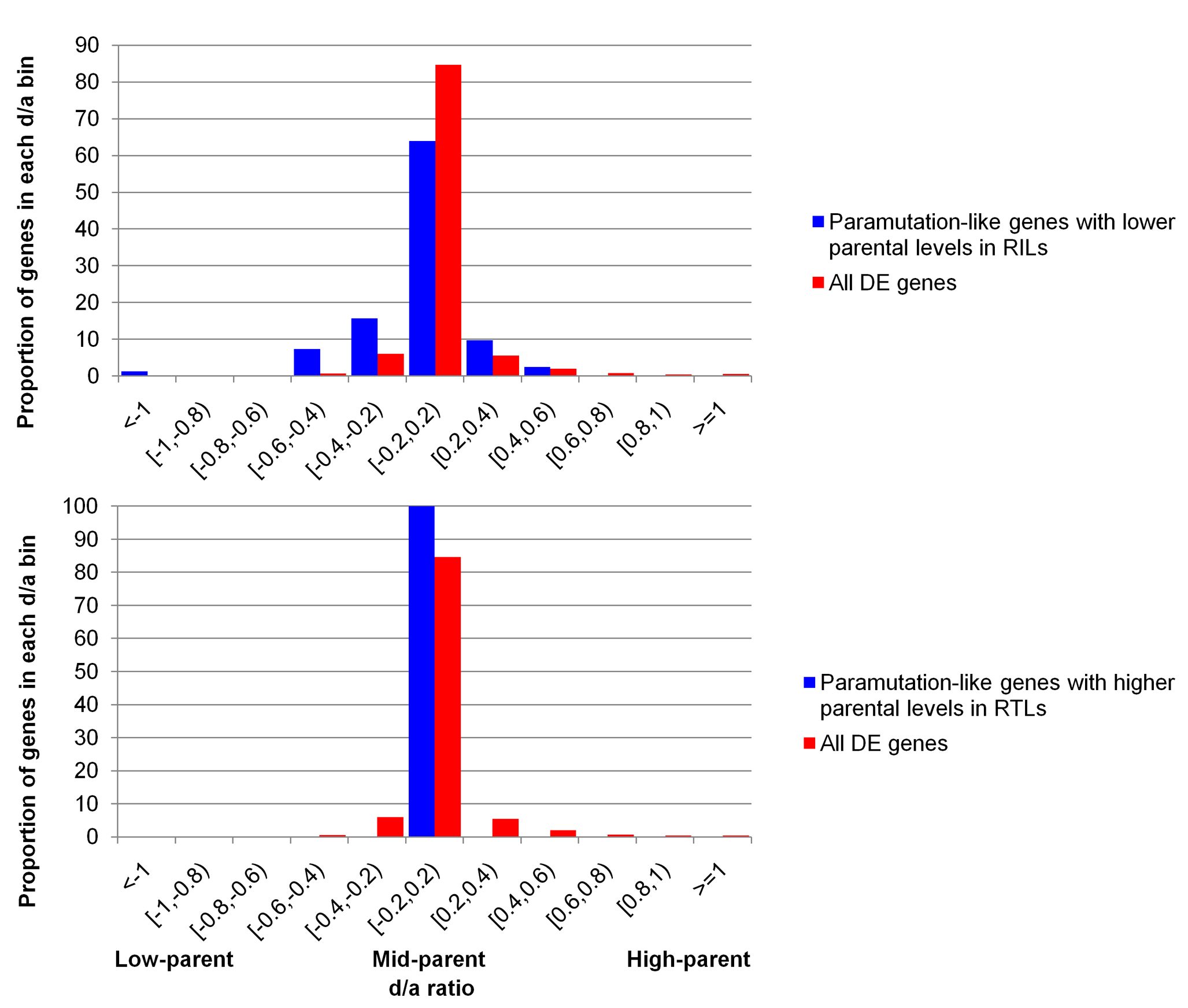

Supplement: Figure S3 — Distribution of d/a values for all differentially expressed genes (2-fold changes) and genes with a paramutation-like pattern. (A) Distributions of d/a ratios in the hybrids and the two parents for paramutation-like genes with lower parental expression level in the RILs. (B) Distributions of d/a ratios in the hybrid and the two parents for paramutation-like genes with higher parental expression level in the RILs. The d/a values represented here indicate the hybrid expression levels relative to the low-parent and high-parent levels. In total, 63 of these paramutation-like genes showed dominant expression patterns in the hybrids (B73×Mo17 and Mo17×B73), in which the genes were expressed at the levels close to one of the parents but significantly different (P<0.05) from the other parent. (TIF) [file pgen.1003202.s003.tif]

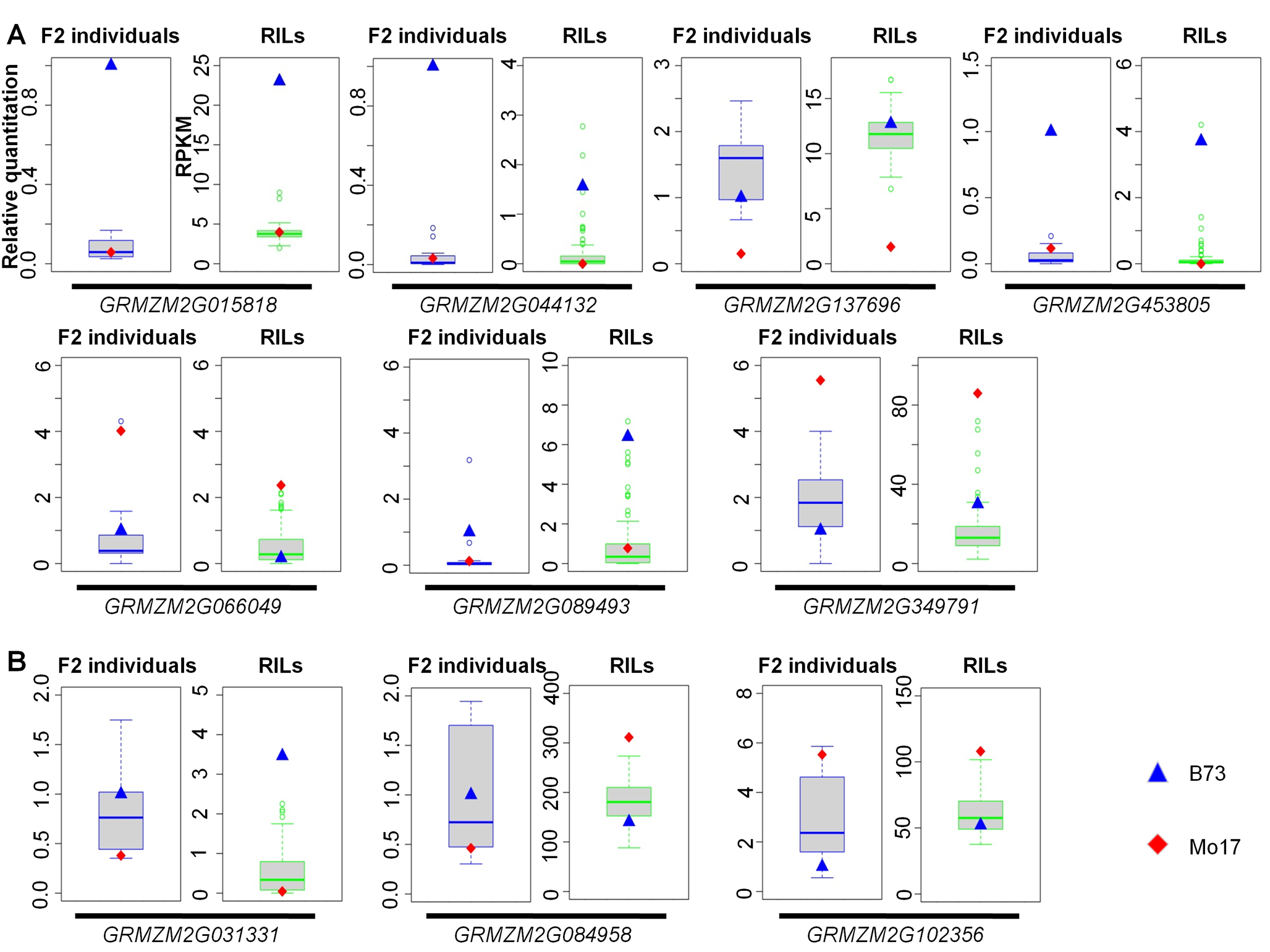

Supplement: Figure S4 — Distribution of expression levels in F2 individuals and RILs for ten genes with paramutation-like expression patterns. For each gene shown along the x-axis, the two y-axes show the expression level for the relative quantitation values in the F2 individuals by qRT-PCR and the RPKM value for the normalized expression level by RNA-seq in the RILs. The blue triangle represents B73, while the red diamond indicates Mo17. (A) Seven (GRMZM2G015818, GRMZM2G044132, GRMZM2G137696, GRMZM2G453805, GRMZM2G066049, GRMZM2G089493 and GRMZM2G349791) of the 10 paramutation-like genes (70%) exhibited similar expression patterns in the F2 individuals as observed in the RILs. (B) Three genes (GRMZM2G031331, GRMZM2G084958 and GRMZM2G102356) did not exhibit paramutation-like expression patterns in the F2 individuals. (TIF) [file pgen.1003202.s004.tif]

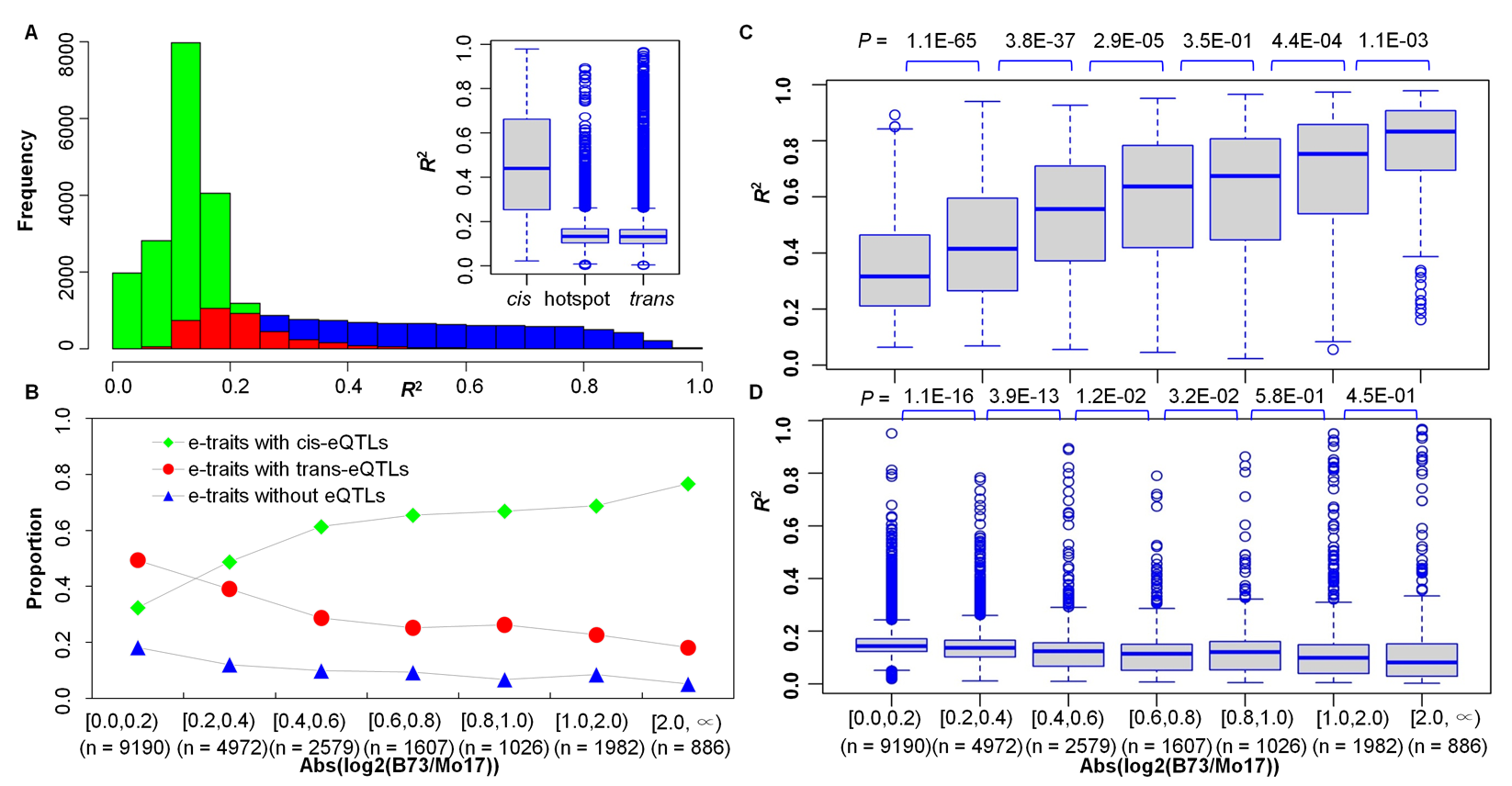

Supplement: Figure S5 — Characteristics of cis-eQTL and trans-eQTL. (A) Shows the R 2 frequency distribution of cis-eQTL and trans-eQTL. Green bars represent trans-eQTL, blue bars show cis-eQTL and red area is the overlap in the graph between cis-eQTL and trans-eQTL. The boxplot shows the R 2 comparison among cis-eQTLs, trans-eQTLs, and trans-eQTLs in trans-eQTL hotspots. In (B), (C) and (D), the x-axis is the absolute value of log2 of expression-level in B73 divided by the level in Mo17. (B) The relationship between the proportion of e-trait distribution and the parental difference. (C) The relationship between R 2 variation of cis-eQTLs and the parental difference. The y-axis in graph (B) shows the R 2 value of cis-eQTLs. (D) The relationship between R 2 variation of trans-eQTLs and the parental difference. The y-axis shows the R 2 value of trans-eQTLs. (TIF) [file pgen.1003202.s005.tif]

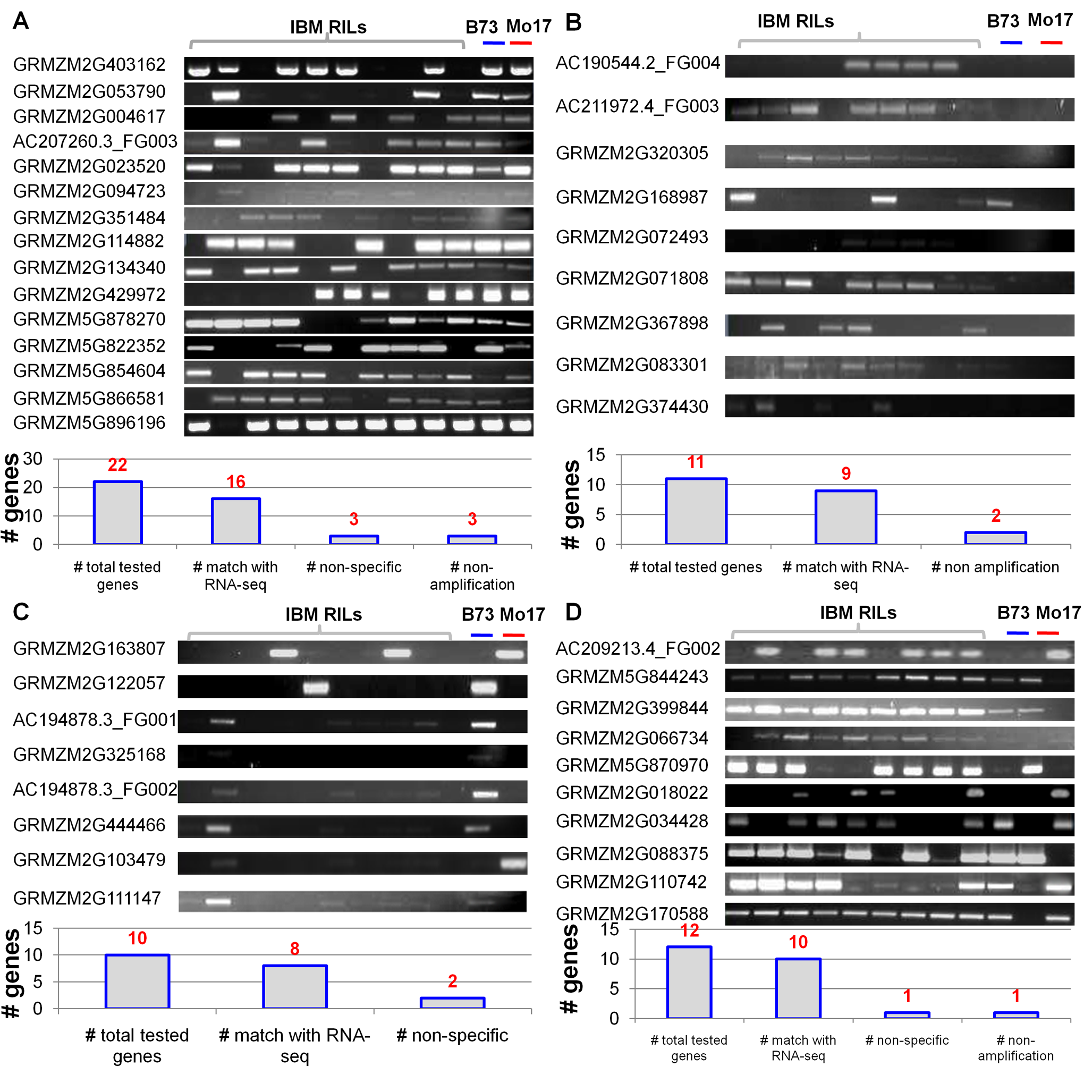

Supplement: Figure S6 — RT-PCR validation of randomly-selected genes with unexpected expression patterns. RT-PCR was conducted for a set of genes with unexpected expression patterns using a subset (10) of the same RILs used for RNA-seq but grown in an independent experiment. All RT-PCR assays were conducted with Touch-Down PCR programs of 35 PCR amplification cycles. Graphs (A), (B), (C) and (D) show the validation of genes with Type I, Type II and Type IIIA and Type IIIB patterns, respectively. The type I pattern represents genes that were expressed in both parents but were not detected (RPKM = 0) in over 10% of the RILs. The type II pattern shows genes that were not detected (RPKM = 0) in the parents but were detected in at least 10% of the RILs. The type III patterns include genes that were expressed in one parent but not the other and had expression in very few RILs (type IIIA) or the majority of the RILs (type IIIB). (TIF) [file pgen.1003202.s006.tif]

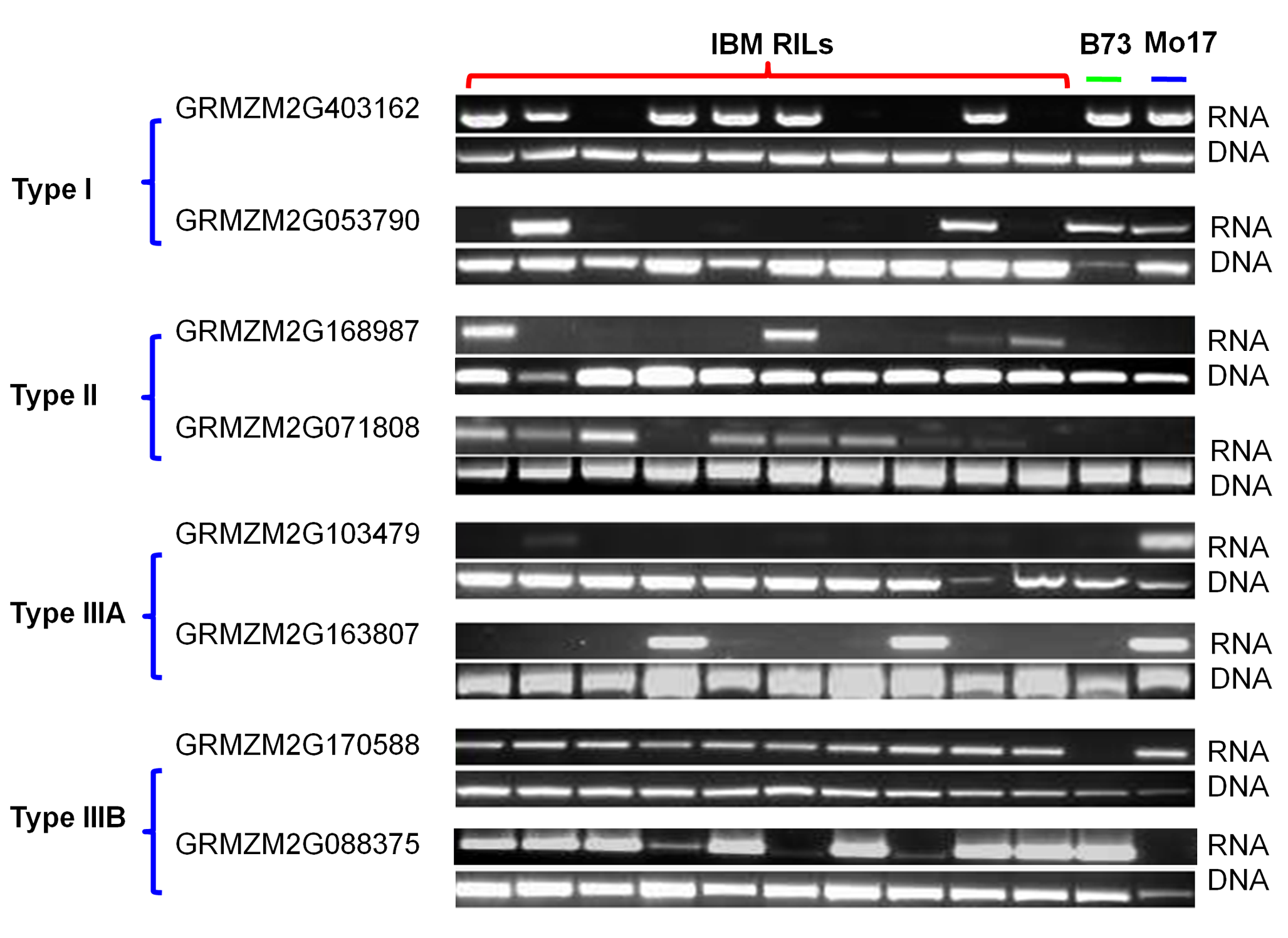

Supplement: Figure S7 — The genes with unexpected segregation for expression are present in the genomic DNA of all samples. PCR was performed on genomic DNA and RT-PCR was performed on RNA for a subset of genotypes for eight genes with unexpected expression patterns. All eight genes were detected in the genomic DNA of all samples but exhibit segregation for gene expression. All RT-PCR and genomic PCR assays were conducted using the Touch-Down PCR program with 35 cycles. (TIF) [file pgen.1003202.s007.tif]

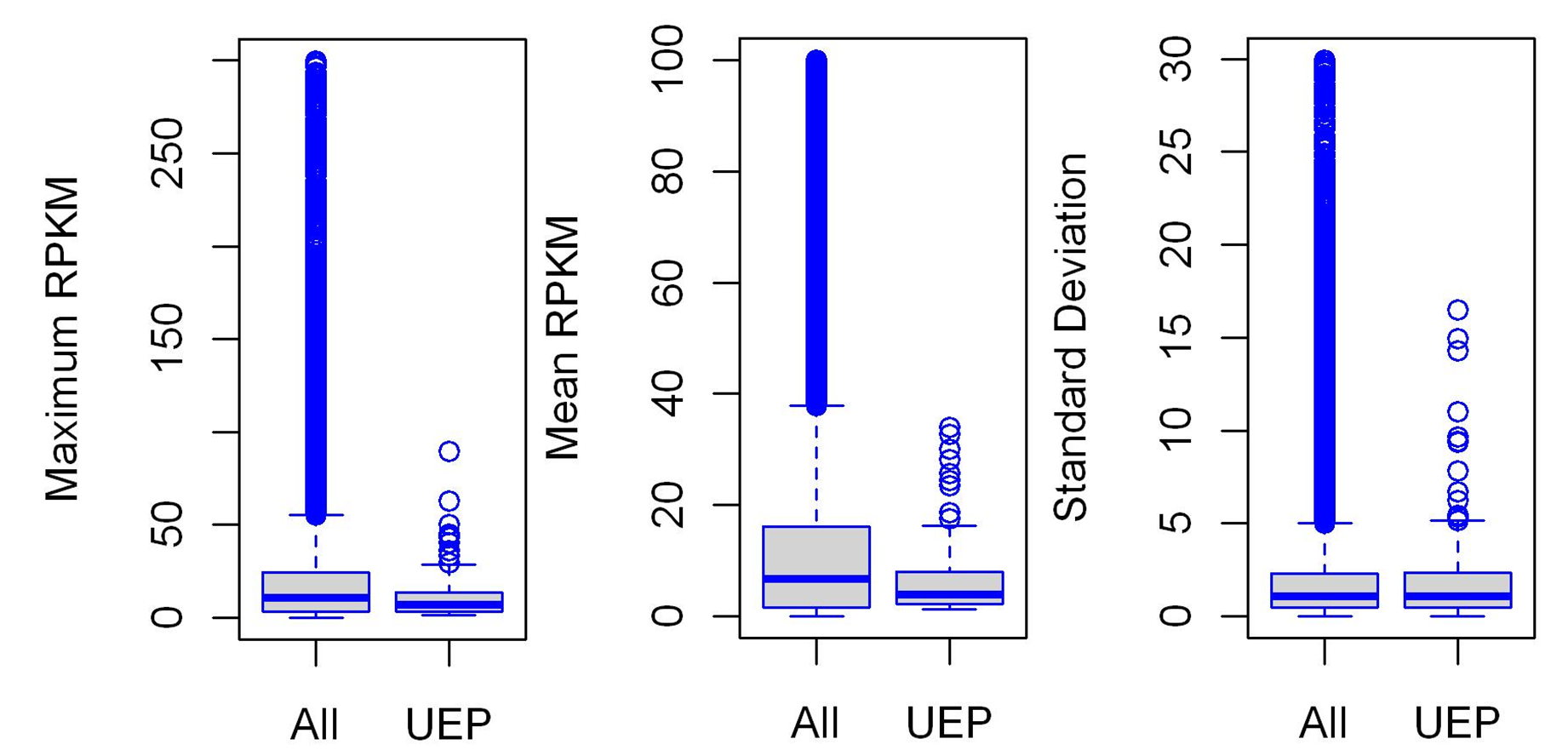

Supplement: Figure S8 — The expression levels and standard deviations of genes with unexpected expression patterns compared with those of all other expressed genes. The genes with unexpected expression patterns (UEP) exhibited the same expression levels and standard deviations as all (All) other expressed genes in the RILs. (TIF) [file pgen.1003202.s008.tif]

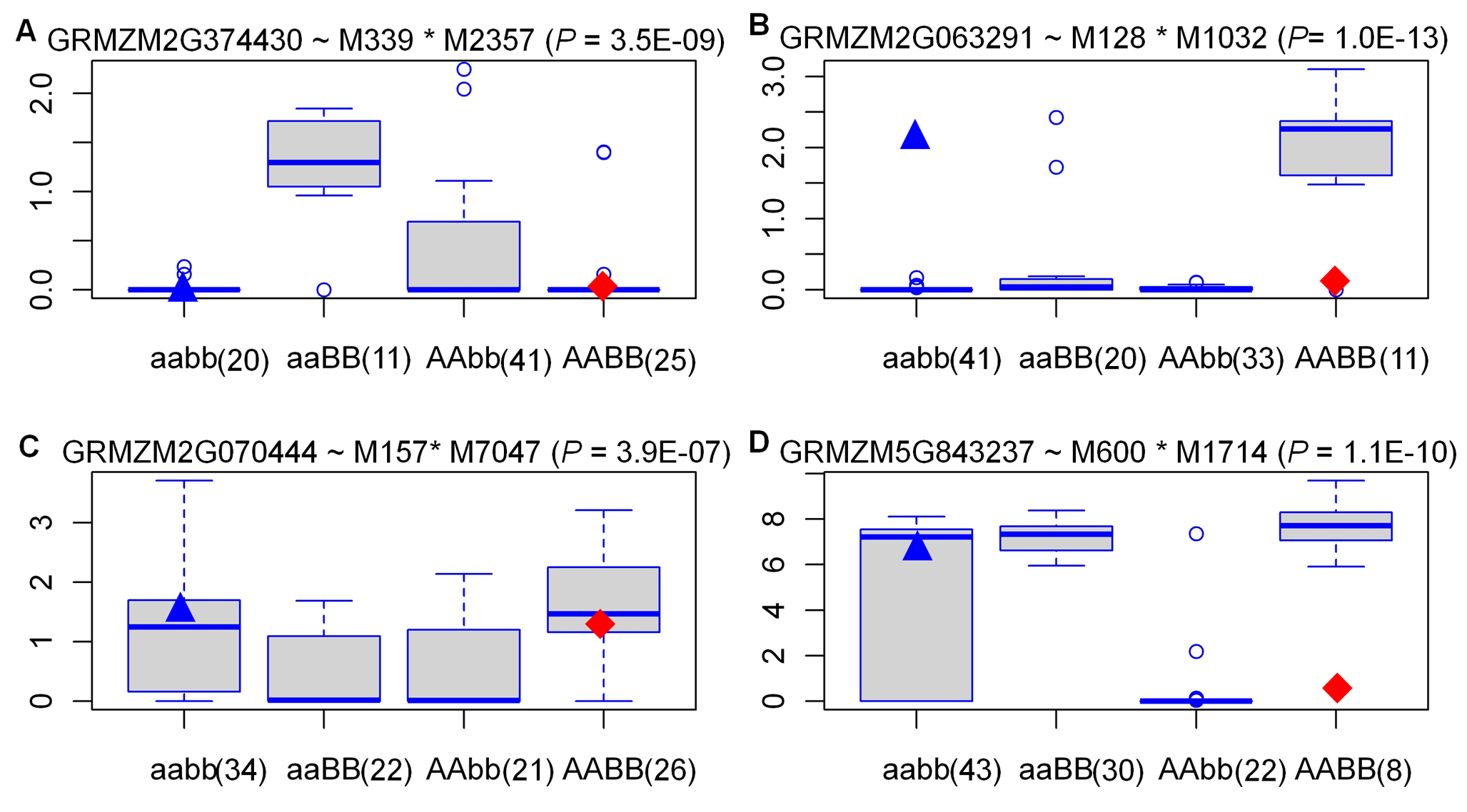

Supplement: Figure S9 — Examples of genes with unexpected expression patterns controlled by two-locus interactions. The x-axis represents different types of genotypes of the RILs. A and B indicate two independent loci, AABB represents the Mo17 genotype, while aabb shows the B73 genotype. The y-axis indicates the normalized expression levels of the RILs and their parents. The blue triangle indicates the expression level in B73, while the red diamond indicates the expression level in Mo17. (A) and (B) show that these genes with expression in only ∼25% of the RILs could be explained by a two locus interaction, while (C) and (D) represent genes that exhibit expression in ∼75% of the RILs and could also be controlled by a two locus interaction. (A), (B), (C) and (D) represent multiple locus interactions for the expression patterns of Type II, Type IIIA, Type I and Type IIIB, respectively. Taken together, 91% of genes with expression in only ∼25% or 75% of the RILs were identified to be controlled by pair-wise locus interactions. (TIF) [file pgen.1003202.s009.tif]

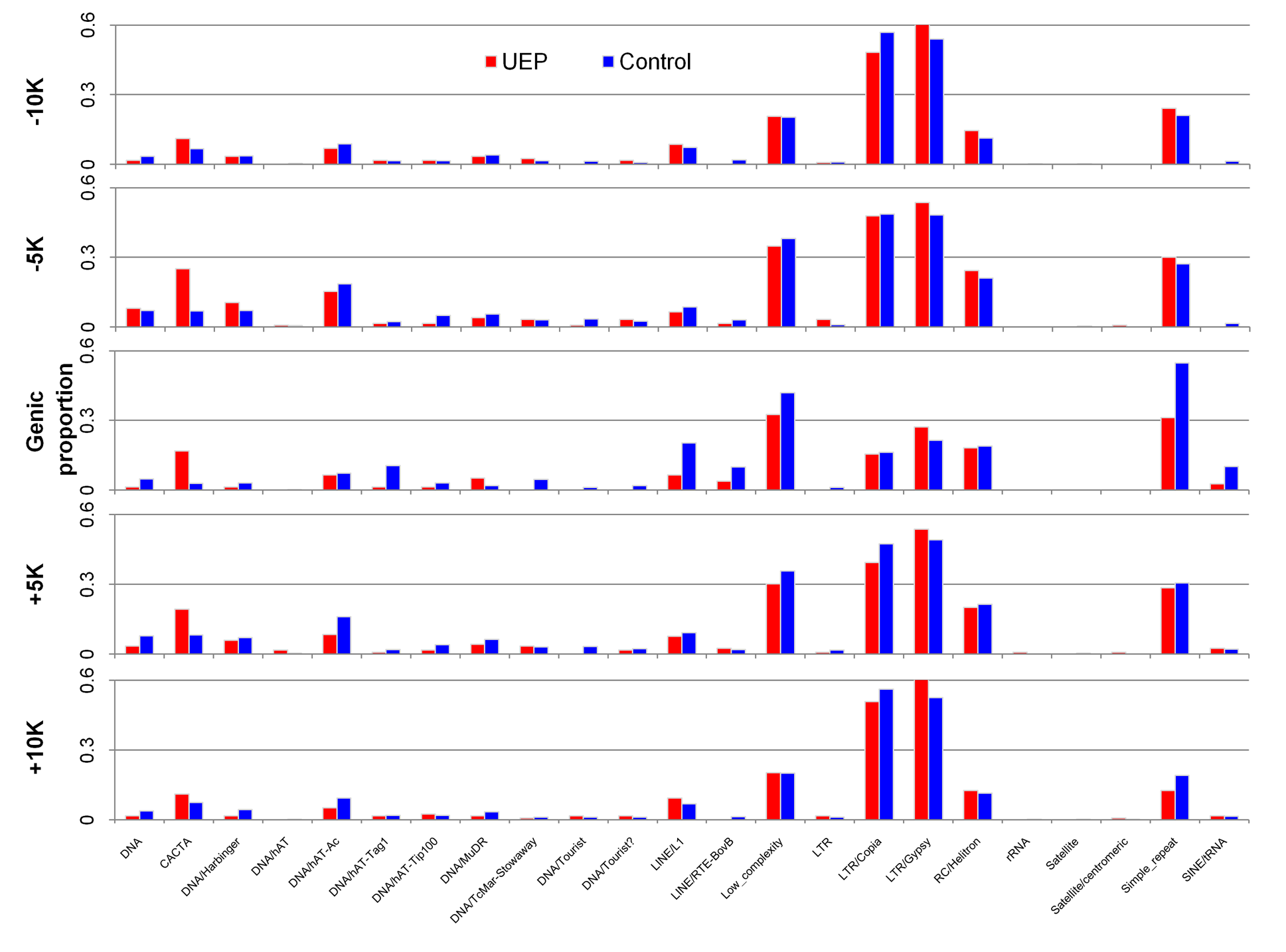

Supplement: Figure S10 — Schematic diagram of the proportion of genes with different transposons in the flanking genomic regions. The x-axis represents different transposons, while the y-axis shows different flanking genomic blocks (5 Kb/block), of which the minus (−) and plus (+) indicate the upstream from the transcriptional start site of the gene and the downstream region from the transcriptional terminal site of the gene, respectively. “UEP” represents the genes with unexpected expression patterns, whereas “Control” shows the randomly-selected genes from the filtered-evidence gene set [2]. (TIF) [file pgen.1003202.s010.tif]
